# Toward Eradication of B-Vitamin Deficiencies: Considerations for Crop Biofortification

**DOI:** 10.3389/fpls.2018.00443

**Published:** 2018-04-06

**Authors:** Simon Strobbe, Dominique Van Der Straeten

**Affiliations:** Laboratory of Functional Plant Biology, Department of Biology, Ghent University, Ghent, Belgium

**Keywords:** micronutrients, biofortification, metabolic engineering, folate, pyridoxine, thiamine, crop improvement, plant development

## Abstract

‘Hidden hunger’ involves insufficient intake of micronutrients and is estimated to affect over two billion people on a global scale. Malnutrition of vitamins and minerals is known to cause an alarming number of casualties, even in the developed world. Many staple crops, although serving as the main dietary component for large population groups, deliver inadequate amounts of micronutrients. Biofortification, the augmentation of natural micronutrient levels in crop products through breeding or genetic engineering, is a pivotal tool in the fight against micronutrient malnutrition (MNM). Although these approaches have shown to be successful in several species, a more extensive knowledge of plant metabolism and function of these micronutrients is required to refine and improve biofortification strategies. This review focuses on the relevant B-vitamins (B1, B6, and B9). First, the role of these vitamins in plant physiology is elaborated, as well their biosynthesis. Second, the rationale behind vitamin biofortification is illustrated in view of pathophysiology and epidemiology of the deficiency. Furthermore, advances in biofortification, via metabolic engineering or breeding, are presented. Finally, considerations on B-vitamin multi-biofortified crops are raised, comprising the possible interplay of these vitamins *in planta*.

## Introduction

In an era of tremendous technological capabilities, insufficient accessibility to nutritious food, a primary human need, still affects over two billion people on a global scale (Bailey et al., 2015; Gupta, 2017). Though approximately 800 million people endure energy deficit due to inadequate amounts of calories in their diet (Haddad et al., 2016; FAOSTAT, 2017), the relative abundance of undernourishment has dropped to almost half in the last 25 years. Unfortunately, the degree of undernourishment witnessed over the last decades has recently passed a minimum, as undernourishment is estimated to have affected 815 million people in 2016, as opposed to 777 million in 2015 (FAOSTAT, 2017). The general trend of a decrease in global undernourishment can be mainly attributed to yield improvement of important staple crops such as rice, maize and wheat, which has more than doubled since 1960 (Long et al., 2015). Unfortunately, caloric malnutrition represents only a portion of the food-related burden of diseases, as micronutrient malnutrition (MNM) is present in over one–fourth of the world’s population (Bailey et al., 2015; Blancquaert et al., 2017; De Lepeleire et al., 2017).

Micronutrient malnutrition comprises shortage of dietary micronutrients, including minerals (iron, zinc, iodine, selenium, etc.) as well as vitamins (Bailey et al., 2015). Micronutrient malnourishment, commonly referred to as the “hidden hunger,” is known to induce diseases and disorders in many populations, not particularly confined to the developing world. Pregnant women and young children are most vulnerable for MNM, often resulting in death (Bailey et al., 2015; De Steur et al., 2015; Win, 2016; De Lepeleire et al., 2017). MNM can be considered an urgent global concern, persistent in many populations and remaining largely hidden (Ruel-Bergeron et al., 2015). Anemia, a condition of suboptimal hemoglobin level (Scott et al., 2014), illustrates the disastrous impact of combined micronutrient shortage on human physiology, as its occurrence has been linked with deficiency in iron (Camaschella, 2015), pro-vitamin A (Semba et al., 1992; West et al., 2007), thiamin (vitamin B1) (Franzese et al., 2017), pyridoxine (vitamin B6) (Clayton, 2006; Hisano et al., 2010) and folate (vitamin B9) (Moll and Davis, 2017). Anemia is held responsible for almost 2 million deaths of children under 5 years old on a yearly basis (Scott et al., 2014), and is estimated to affect more than 2 billion people globally. It is estimated that half of the cases of anemia could be attributed to deficiency in one or more micronutrients, though primary factors are iron and folate shortage (Scott et al., 2014).

High incidence of micronutrient deficiencies are, in many cases, related to monotonous diets, largely consisting of energy rich, starchy staples (Blancquaert et al., 2017; De Lepeleire et al., 2017). These crops including wheat, rice, potato, cassava, corn and plantain have the tendency to contain inadequate levels of vitamins and therefore expose the population, consuming massive amounts of these staples, to the risk of vitamin deficiencies (**Table 1**). This is a downside of the cheap supply of energy rich staples, which enabled the aforementioned decrease in caloric malnourishment.

**Table 1:** Vitamin content of six major staple crops.

	RDA^1^	Wheat (*Triticum aestivum*) Soft, white	Rice (*Oryza sativa*) White, long-grain, regular, raw	Potato (*Solanum tuberosum*) Flesh and skin, raw	Cassava (*Manihot esculenta*) Raw	Corn (*Zea mays*) Sweet, white, raw	Plantain (*Musa* sp.) Raw
B1 (mg)	1.4	0.41 (sufficient)	0.07 (5)	0.081 (4)	0.087 (3)	0.2 (2)	0.052 (8)
B6 (mg)	2	0.378 (sufficient)	0.164 (3)	0.298 (2)	0.088 (4)	0.055 (9)	0.299 (2)
B9 (μg)	600	41 (3)	8 (16)	15 (8)	27 (4)	46 (3)	22 (8)
Highest consumption (g/capita.day)^2^	/	609 (Azerbaijan)	470^3^ (Bangladesh)	502 (Belarus)	678 (Democratic Republic of the Congo)	434 (Lesotho)	350 (Ghana)

*Vitamin content data were retrieved from the USDA database (USDA, 2016). RDA data were retrieved from (Trumbo et al., 2001). Values represent vitamin content of 100 g fresh edible portion of each crop. Fold enhancement needed to reach the RDA of the corresponding vitamin upon consuming the highest average national serving, is indicated between brackets. Data on staple crop consumption are derived from FAOSTAT (FAOSTAT, 2017). ^*1*^Highest recommended daily allowance (RDA). ^*2*^In 2013. ^*3*^Milled equivalent.*

Given the observation that MNM have a detrimental effect on global human health, there is a great need to strongly reduce these deficiencies, also stated in the Copenhagen consensus, where micronutrient interventions were ranked as the number one priority, related to Sustainable Development Goal 2 (SDG2), requiring great global investment (Copenhagen Consensus, 2012). Fortunately, there are several means to combat MNM in an effective way, which can be divided in education, supplementation and biofortification. Behavioral interventions, consisting of educational efforts encouraging dietary diversification, are the ideal means to improve the micronutrient status of a population (Reinbott et al., 2016). This strategy, however, requires changes in cultural or religious habits of certain communities, as well as recurrent interventions (Blancquaert et al., 2017). Fortification includes the administration of micronutrient to the population under the form of pills or fortification of food products (such as flour). The latter method, which is mandatory in many countries, has proven to be a rapid medium to ensure optimal micronutrient levels in the troubled populations (Pasricha et al., 2014; Sandjaja et al., 2015; Atta et al., 2016; Rautiainen et al., 2016; Wang et al., 2016). Unfortunately, supplementation depends on specialized infrastructure and appears difficult to implement in poor rural populations who have the highest demand for micronutrient interventions (Blancquaert et al., 2017). Luckily, biofortification, which involves the augmentation of the natural nutritional value of crops, can be addressed as a valuable additional strategy in the battle against MNM (Blancquaert et al., 2017; Garcia-Casal et al., 2017; Martin and Li, 2017; Strobbe and Van Der Straeten, 2017). Biofortification of locally consumed crops does not require changes in consumer behavior and demands only a one-time investment (De Steur et al., 2015, 2017; Bouis and Saltzman, 2017). Biofortification of staple crops, massively consumed in deficient populations, is an excellent way to supply sufficient micronutrients (Blancquaert et al., 2017; De Lepeleire et al., 2017). Biofortification *sensu stricto*, thereby omitting agricultural interventions (Cakmak and Kutman, 2017; Watanabe et al., 2017), comprises breeding techniques as well as genetic engineering approaches (Blancquaert et al., 2017; Bouis and Saltzman, 2017). Breeding strategies have the advantage to be easily implemented in agriculture, as they do not require exhaustive regulations (Mejia et al., 2017). However, the scope of the breeding approaches is confined to sexual compatibility, thereby lacking the ability to exploit useful animal or prokaryotic derived characteristics (Strobbe and Van Der Straeten, 2017). Biofortification via metabolic engineering, overrules this restriction. Furthermore, the latter approaches enable creation of a biofortification strategy blue-print, applicable to a wide variety of food crops (Strobbe and Van Der Straeten, 2017). Metabolic engineering does, however, require a great knowledge of the specific micronutrient metabolism and its importance in the physiology of the plant.

This review reflects upon the acquired knowledge which enabled successful B-vitamin biofortification in food crops, bundling information on thiamin (B1), pyridoxine (B6), and folates (B9). This evaluation includes vitamin function in plant growth and development as well as importance in human pathophysiology, epidemiology and accomplishments in biofortification. Furthermore, in view of multi-biofortification, the simultaneous biofortification of multiple vitamins and minerals, possible synergistic or adverse effects of micronutrient combinations, are scrutinized. Multi-biofortification endeavors are the step-stone for future eradication of MNM. A list of abbreviations can be found in **Supplementary Table S1**.

## Vitamin B1 – Thiamin

**FIGURE 1:**
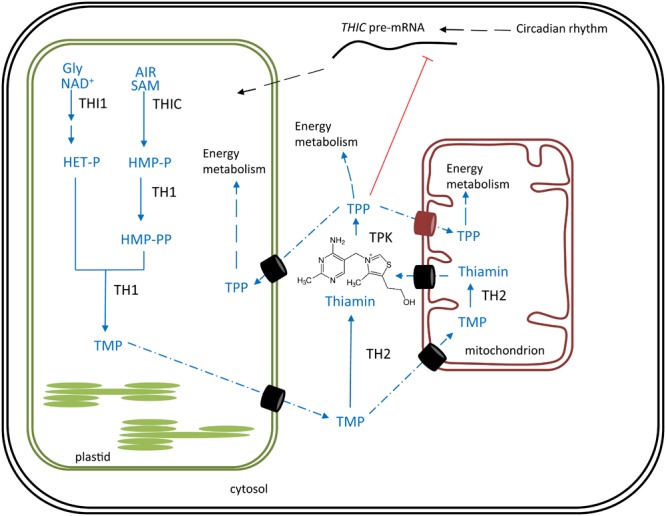
Thiamin biosynthesis in plants. Synthesis of pyrimidine and thiazole moieties as well as their condensation occurs in plastids. Biosynthesis pathway is shown in blue, enzymes in black. Transport across membranes is proposed to be carrier-mediated (barrels), of which the identified mitochondrial TPP carrier is indicated (red barrel) (Frelin et al., 2012). The chemical structure of thiamin is depicted, of which the free hydroxyl group can be pyrophosphorylated by action of thiamin pyrophosphokinase (TPK). End product feed-back, performed by TPP on the *THIC* riboswitch, is depicted in red. Products: Gly, glycine; NAD^+^, nicotinamide adenine dinucleotide; SAM, *S*-adenosylmethionine; AIR, 5-aminoimidazole ribonucleotide; HET-P, 4-methyl-5-β-hydroxyethylthiazole phosphate; HMP-P, 4-amino-2-methyl-5-hydroxymethylpyrimidine phosphate; HMP-PP, HMP-pyrophosphate; TMP, thiamin monophosphate; TPP, thiamin pyrophosphate. Enzymes: THIC, HMP-P synthase; THI1, HET-P synthase; TH1, HMP-P kinase/TMP pyrophosphorylase; TPK, thiamin pyrophosphokinase; TH2, TMP phosphatase, PALE GREEN1.

Thiamin is a water soluble B-vitamin consisting of a pyrimidine ring, linked to a thiazole moiety by a methylene bridge (Lonsdale, 2006) (**Figure 1**). Vitamin B1 consists of different itamer forms of thiamin, predominantly occurring as thiamin and its different phosphate esters thiamin pyrophosphate (TPP) and thiamin monophosphate (TMP). However, other forms of thiamin do exist, such as thiamin triphosphate, though their contribution to the total pool is rather marginal (Gangolf et al., 2010). In literature, thiamin(e) is sometimes confusingly used to describe the total pool of the different B1-vitamers, here simply referred to as B1. The bioactive vitamer is TPP, serving as cofactor in multiple enzymatic reactions.

### Biosynthesis

Biosynthesis of TPP in plants takes place in plastids, cytosol and mitochondria (**Figure 1**). Although the TMP vitamer is synthesized in the chloroplast, subsequent enzymatic reactions in mitochondria and cytosol are required to finalize the *de novo* biosynthesis of TPP, the bioactive vitamer (Mimura et al., 2016; Goyer, 2017; Hsieh et al., 2017). Formation of TMP involves the synthesis of the thiazole and pyrimidine moieties, followed by their condensation, all of which occur in chloroplasts (Goyer, 2017). The thiazole moiety of the thiamin structure is created by the action of 4-methyl-5-β -hydroxyethylthiazole phosphate (HET-P) synthase (THI1), requiring NAD^+^ and glycine as a substrate and yielding HET-P (Godoi et al., 2006). In this reaction the THI1 enzymes is consumed, as it supplies a sulfide from a cysteine residue, making THI1 a ‘suicidal’ enzyme. 4-amino-2-methyl-5-hydroxymethylpyrimidine phosphate (HMP-P) synthase (THIC) is an iron-sulfur cluster containing enzyme, catalyzing the formation of HMP-P, the pyrimidine intermediate of thiamin biosynthesis (Raschke et al., 2007; Kong et al., 2008; Goyer, 2017). This reaction requires 5-aminoimidazole ribonucleotide (AIR) (derived from purine metabolism) and *S*-adenosylmethionine (SAM) as substrates (Chatterjee et al., 2008). This is the key regulatory step of thiamin biosynthesis, as the promotor is under control of the circadian clock, and the terminator is feedback inhibited by the end product TPP (Bocobza et al., 2013). This terminator contains a riboswitch, rarely seen in eukaryotes (Wachter et al., 2007). The riboswitch acts by binding TPP in its pre-mRNA state, resulting in the formation of an unstable splice variant of the *THIC* gene, thereby causing a lowered THIC activity (Wachter et al., 2007; Bocobza et al., 2013). Next, the bifunctional enzyme harboring HMP-P kinase and TMP pyrophosphorylase activities (TH1), catalyzes the HMP-P phosphorylation and subsequent condensation of HMP-PP and HET-P to form TMP (Ajjawi et al., 2007b). TMP forms the end product of plastidial thiamin biosynthesis and is further processed by the PALE GREEN1/TH2 enzyme, after exiting the plastids (Mimura et al., 2016; Hsieh et al., 2017). The subcellular localization of TH2 action, being cytosolic, mitochondrial, or both, has been debated. First, the enzyme was discovered in the cytosolic fraction of Arabidopsis (Ito et al., 2011; Mimura et al., 2016). Later experimental evidence, utilizing translational fusions with green fluorescent protein (GFP), confirmed the cytosolic location of the TH2 enzyme, realized from a (preferred) native secondary translational initiation site (Mimura et al., 2016). This secondary translational initiation site yields a protein which lacks the functional N-terminal mitochondrial targeting peptide of TH2. Therefore, TH2 was considered to be predominantly residing in the cytosol though likely also present in mitochondria. More recently, TH2 was found to be almost exclusively localized in the mitochondria of *th2/pale green1* rescued with a GFP-fused TH2, controlled by the cauliflower mosaic virus (CaMV) 35S promoter (Hsieh et al., 2017). These seemingly contradicting findings can be explained by the fact that a strong constitutive promoter (35S) preceding the full coding sequence of *TH2*, favors the first translational start site and incorporates the mitochondrial targeting peptide into the arising protein (Hsieh et al., 2017). However, these findings highlight the ability of sole mitochondrial TH2 activity to complement the *th2/pale green1* mutant, indirectly hinting at the existence/necessity of a mitochondrial TMP importer as well as a thiamin exporter. Combining these results, it can be concluded that TMP dephosphorylation, executed by TH2, likely occurs primarily in the cytosol and to a lesser extent in mitochondria. However, it cannot be excluded that this subcellular localization of TH2 action might change in different conditions/tissues/species. The reaction mediated by TH2 yields thiamin. In turn, thiamin, is the substrate for thiamin pyrophosphokinase (TPK), producing the active vitamer TPP in the cytosol (Ajjawi et al., 2007a). TPP subsequently travels to the different subcellular locations via carriers. Two mitochondrial TPP carriers have been partially characterized in plants (Frelin et al., 2012).

### Role in Plant Physiology

Vitamin B1, sometimes called the ‘energy vitamin,’ plays a crucial role in plant energy homeostasis, mostly by the role of TPP as a cofactor (Goyer, 2010). TPP is a cofactor for three enzymes which are central to the energy metabolism. First, the pyruvate dehydrogenase (PDH) complex, catalyzing pyruvate decarboxylation, yielding acetyl CoA and NADH, necessary for the tricarboxylic acid (TCA or Krebs) cycle and biosynthetic processes, respectively (Bocobza et al., 2013). In addition, in the tricarboxylic acid cycle, α-keto-glutarate dehydrogenase (2-oxoglutarate dehydrogenase E1 component, OGDH) functioning also requires TPP, further accentuating its critical role in central metabolism (Rapala-Kozik et al., 2012). Third, TPP is an essential cofactor of the transketolase (TK) enzyme, playing a key role in the Calvin cycle as well as the pentose phosphate pathway, which renders pentose sugars as well as NADPH to the cell (Goyer, 2010).

Hence B1, in its form of TPP, controls a few key steps in central aerobic energy metabolism. In this regard, TPP has been suggested to influence the flux through these pathways, as it is required in their rate-limiting steps (Bocobza et al., 2013). Indeed, modestly increasing TPP concentration, through introduction of a non-functional riboswitch in Arabidopsis, induced enlarged activity of TPP-dependent enzymes (PDH, OGDH, and TK) (Bocobza et al., 2013). Moreover, these plants emitted larger amounts of CO_2_, suggesting overactive oxidative metabolism. This is further confirmed by the observation of depleted starch reserves at the beginning of the light period in high TPP lines (Bocobza et al., 2013). This presents a clear rationale behind the strict circadian regulation on B1 biosynthesis. As a consequence of its influence on central metabolism, metabolite composition, particularly that of amino acids, is severely altered in plants with aberrant B1 composition (Bocobza et al., 2013).

B1 metabolism was shown to have a clear function in enabling plants to cope with biotic as well as abiotic stresses. Thiamin biosynthesis as well as B1 levels were observed to increase upon application of abiotic stresses such as high light, drought, salt and oxidative stress, conferring tolerance (Kaya et al., 2015; Yee et al., 2016). Remarkably, this B1-induced tolerance to oxidative stress was concomitant with decreased production of reactive oxygen species (ROS) (Tunc-Ozdemir et al., 2009). The exact molecular basis for this role of B1 in stress adaptation of plants remains partly unknown. Given the enhanced expression of TPP-dependent enzymes in plants exposed to drought stress, the influence of B1 on abiotic stress control seems to be via its end product TPP (Rapala-Kozik et al., 2012). On the other hand, the B1 biosynthesis enzyme THI1, responsible for plastidial thiazole biosynthesis, appears to be able to directly regulate stomatal closure (Li et al., 2016). The potential of B1 to enhance abiotic stress tolerance was, however, not observed in engineered Arabidopsis lines (Dong et al., 2015). Considering biotic stresses, B1 has been shown to confer systemic acquired resistance (SAR) (Ahn et al., 2007; Bahuguna et al., 2012; Boubakri et al., 2012). Thiamin-treated plants depicted enhanced ROS (hydrogen peroxide, produced upon up-regulation of superoxide dismutase) accumulation upon infection (Bahuguna et al., 2012), contrasting their role of decreasing ROS in abiotic stresses (Tunc-Ozdemir et al., 2009; Kaya et al., 2015). By doing so, thiamin provokes priming, a state in which the plant has the ability to react more rapidly upon infection (Conrath et al., 2006; Ahn et al., 2007). This priming effect, described for B1, was confirmed in high B1 engineered Arabidopsis (Dong et al., 2015), but not seen in rice (Dong et al., 2016). Moreover, thiamin treatment of plants induced higher accumulation of phenolic compounds, salicylic acid (SA) (through higher phenylalanine ammonia lyase activity) and nitrogen assimilation (via increased nitrate reductase activity) (Bahuguna et al., 2012).

### Pathophysiology and Epidemiology

The central role of B1 in central (oxidative) metabolism in humans is reflected in its pathophysiology upon vitamin deficiency. TPP plays an indispensable role in energy metabolism as a cofactor in cleavage of α-keto acids (Adeva-Andany et al., 2017), as well as general oxidative metabolism, identical to its role *in planta*. While having lost the ability to synthesize thiamin during their evolution, humans possess the potential to interconvert the different thiamin phosphate-esters (Zhao et al., 2001; Banka et al., 2014).

B1 was the first vitamin for which deficiency was characterized, as it was considered a “vital amine’ (hence ‘vitamine’), defined as a substance inducing the disease beriberi upon insufficient consumption (Lonsdale, 2006). Beriberi is a disease occurring upon severe B1 deficiency, divided in wet and dry beriberi, depending on whether it is manifested in the cardiovascular system or in the peripheral nervous system, respectively (Abdou and Hazell, 2015). B1 deficiency can cause heart problems, and even lead to heart failure (Roman-Campos and Cruz, 2014). Different symptoms, such as enlarged heart and increased venous pressure, have been reported. B1 deficiency has also been linked to Sudden Infant Death Syndrome (SIDS), due to brainstem malfunctioning related to hypo-oxidative metabolism (Lonsdale, 2015). An insufficient supply of the B1-vitamin can induce severe alternation of the nervous system, which leads to a disorder called Wernicke’s encephalopathy (WE) (Jung et al., 2012). WE involves the arising of selective brain lesions, the first symptoms of which include confusion, apathy and impaired awareness, eventually ending in coma and death. B1 deficiency-induced disorders are in many cases easily reverted with thiamin application, and often witnessed in patients suffering from chronic alcoholism (Butterworth, 1993). The detrimental effect of B1-deficiency on brain functioning can be explained by the strong dependency of the brain on the oxidative metabolism (Butterworth, 1993; Gibson et al., 2005).

One of the greatest risks of B1-deficiency, along with the lethal consequences of untreated WE, is the difficulty of diagnosis, leaving many illnesses untreated (Harper, 2006). Although cases of severe beriberi have become rare, outbreaks of B1-deficiency-induced beriberi have been reported on a global scale, causing many deaths, even upon sufficient access to healthcare (Luxemburger et al., 2003; Ahoua et al., 2007). Hence, in developing countries, B1-deficiency often is not linked to the observed casualties (Barennes et al., 2015). Moreover, infantile exposure to B1-deficiency was recently shown to have long-term effects on motor functions and balance of the child (Harel et al., 2017). Furthermore, elderly people have been shown to be highly susceptible to B1-deficiency, even in the developed world (Hoffman, 2016). Indeed, an investigation in New York state (United States) identified 14% of elderly as being B1-deficient (Lee et al., 2000). B1-deficiency is likely to be exacerbating Alzheimer’s disease, and could therefore be considered a serious threat, definitely not confined to the developing world (Gibson et al., 2013).

Good sources of vitamin B1 are, besides animal-derived products (meats, liver, eggs, and dairy products), beans and peas, nuts and whole grains (Lonsdale, 2006; USDA, 2016). Different massively consumed crops, such as rice, cassava, potato and plantain contain inadequate amounts of B1 (**Table 1**). In the case of rice, polishing, which removes the aleurone layer to avoid rancidification, eliminates many nutritionally valuable substances, including B1 (Goyer, 2017). This is illustrated by the original observation of B1-deficiency induced paralysis and death in fowls fed with polished rice, reversible by administration of the rice polishings (Lonsdale, 2006). Therefore, overconsumption of such staples in a monotonous diet, imposes a serious threat to human health. Furthermore, high carbohydrate intake increases the need of dietary B1, which is explained by its role in carbohydrate catabolism (Elmadfa et al., 2001). This emphasizes the need for increased B1 levels in these popular starchy crops.

### Biofortification

Engineering of the thiamin biosynthesis pathway to augment thiamin content in plants has been attempted recently (Dong et al., 2015, 2016). The key step in thiamin -and therefore B1- engineering is the first committed step in plastidial pyrimidine biosynthesis, *THIC* (Raschke et al., 2007). Activity of the THIC enzyme seems to be a major determinant of B1 biosynthesis, as indicated by the oscillations of the corresponding mRNA transcript with TMP levels (Bocobza et al., 2013). Moreover, this gene harbors a TPP-binding riboswitch in its 3′ UTR, which enables it to destabilize its mRNA upon high TPP prevalence (Wachter et al., 2007). This feedback mechanism, rather unique in eukaryotes, further highlights THIC as a regulatory point in B1 biosynthesis and therefore the *THIC* gene as an ideal candidate in metabolic engineering approaches (Pourcel et al., 2013). Indeed, eliminating this riboswitch, thereby removing the feedback inhibition on THIC, elevates thiamin level 1.6-fold in Arabidopsis (Bocobza et al., 2013). Enhancing the flux toward biosynthesis of the pyrimidine intermediate is likely insufficient for accumulation of B1. Indeed, feeding Arabidopsis seedlings with the intermediates pyrimidine and thiazole indicates that both are necessary to achieve higher levels of B1 (Pourcel et al., 2013). Combined overexpression of *THIC* and *THI1*, the plastidial thiazole biosynthetic enzyme (Godoi et al., 2006), further enhanced B1 levels of Arabidopsis over threefold compared to wild type (Dong et al., 2015). Similarly, implementation of this combined engineering strategy in rice resulted in B1 increase of 2.5-fold in leaves and 5-fold in unpolished grains (Dong et al., 2016). However, B1 levels remained barely affected in polished rice seeds. Future engineering strategies in B1-biofortification will tackle additional bottlenecks in B1-accumulation as well as applying engineering strategies to specific tissues (Goyer, 2017). Taken into account the detrimental effects of *THIC*-riboswitch elimination, resulting in chlorotic plants with enhanced carbohydrate oxidation (Bocobza et al., 2013), B1 biofortification should be approached with caution.

Besides metabolic engineering, there are opportunities to enhance B1 content in crops via breeding techniques. Indeed, several (wild) potato varieties were identified which harbor over 2-fold difference in B1 content compared to popular agricultural potato cultivars (Goyer and Sweek, 2011). Similarly, up to 2.7-fold variation was found in different cassava accessions (Mangel et al., 2017). Previously, over 10-fold B1 variation has been measured in rice (Kennedy and Burlingame, 2003). Recently, a genome wide association study (GWAS) identified multiple quantitative trait loci (QTL), underlying B1 content in common wheat (Li et al., 2017). These results imply that breeding strategies could help in acquiring higher B1 levels in popular/regional crop varieties. On the other hand, elevating of B1 levels through exposure to certain biological stresses has been suggested, as this proves to augment B1 biosynthesis by significantly increasing the expression of the biosynthesis genes (Kamarudin et al., 2017).

## Vitamin B6

**FIGURE 2:**
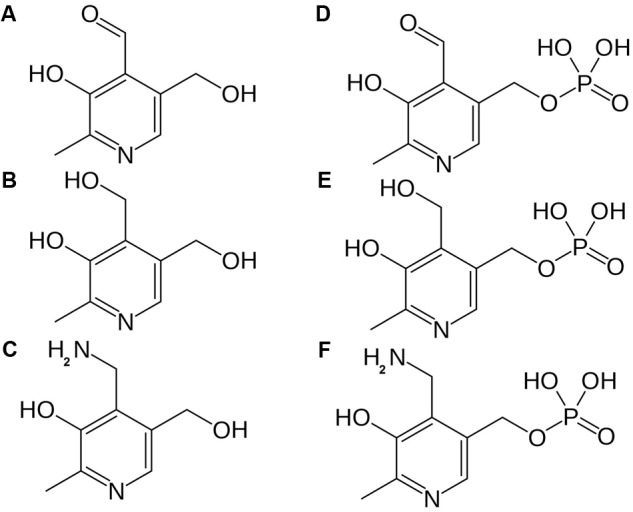
Chemical structure of different B6 vitamers. **(A)** pyridoxal (PL), **(B)** pyridoxine (PN), **(C)** pyridoxamine (PM), **(D)** pyridoxal-phosphate (PLP), **(E)** pyridoxine-phosphate (PNP), **(F)** pyridoxamine-phosphate (PMP).

Vitamin B6 represents a group of water-soluble molecules with similar biochemical properties, consisting of pyridoxine (PN), pyridoxal (PL), pyridoxamine (PM), and their phosphorylated esters (Fudge et al., 2017). PN, PL and PM differ by carrying a hydroxymethyl, an aldehyde or an aminomethyl substituent, respectively (**Figure 2**) (Hellmann and Mooney, 2010). Considering these six vitamers, the phosphorylated pyridoxal (PLP, **Figure 2D**) is the most bioactive, functioning as a cofactor in over a hundred reactions (Fudge et al., 2017). B6 can be considered a powerful antioxidant, comparable to carotenes (vitamin A) and tocopherols (vitamin E), as they are able to quench ROS (Bilski et al., 2000).

### Biosynthesis

**FIGURE 3:**
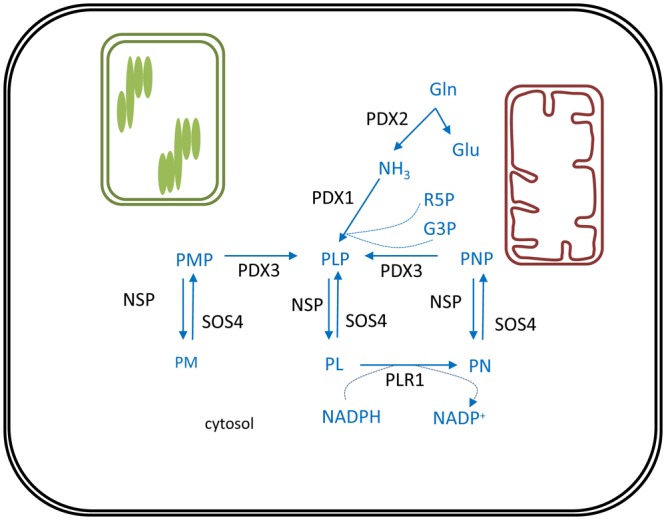
Vitamin B6 biosynthesis in plants. *De novo* biosynthesis of vitamin B6 is cytosolic. PLP, the major bioactive B6 vitamer is synthesized via consecutive action of PDX2 and PDX1 enzymes. Reactions required to interconvert these different vitamers are depicted. Biosynthesis pathway is shown in blue, enzymes in black. Products: Gln, glutamine; Glu, glutamate; NH_3_, ammonia; R5P, ribose 5′-phosphate; G3P, glyceraldehyde 3-phosphate; PL, pyridoxal; PN, pyridoxine; PM, pyridoxamine; PLP, pyridoxal phosphate; PNP, pyridoxine phosphate; PMP, pyridoxamine phosphate; NADPH, nicotinamide adenine dinucleotide phosphate. Enzymes: PDX1, pyridoxal phosphate synthase protein; PDX2, pyridoxine biosynthesis glutaminase; PDX3, PMP/PNP oxidase; SOS4, SALT OVERLY SENSITIVE4; NSP, non-specific phosphatase; PLR1, pyridoxal reductase.

*De novo* biosynthesis of vitamin B6 takes place in the cytosol and comprises only two enzymes (**Figure 3**). Pyridoxal phosphate synthase protein (PDX1) generates pyridoxal 5′-phosphate (PLP) utilizing ammonia, glyceraldehyde 3-phosphate (G3P) and ribose 5′-phosphate (R5P) as substrates (Titiz et al., 2006). This ammonia originates from the reaction catalyzed by the PDX2 glutaminase, which releases ammonia from glutamine to yield glutamate (Tambasco-Studart et al., 2007). Furthermore, PMP/PNP oxidase (PDX3) is considered a crucial step in PLP salvage, ensuring its retrieval from the PMP and PNP vitamers (Sang et al., 2007). The non-phosphorylated vitamers PL, PM, and PN, can be converted to their corresponding phosphorylated vitamers by the action of the SALT OVERLY SENSITIVE 4 kinase (SOS4) (Shi et al., 2002). Finally, a pyridoxal reductase (PLR1) was identified, mediating a NADPH-requiring conversion of PL to PN (Herrero et al., 2011). Through these reactions plants are capable of balancing the different vitamer forms of B6, which is required to ensure controlled growth and development (Colinas et al., 2016).

### Role in Plant Physiology

Vitamin B6 is involved in a plethora of metabolic reactions, serving as cofactor or required as an antioxidant (Tambasco-Studart et al., 2005; Mooney and Hellmann, 2010). PLP is considered to function as a cofactor for about 200 enzymatic reactions in Arabidopsis (Fudge et al., 2017). These PLP-dependent enzymes, covering oxidoreductases, transferases, hydrolases, lyases, and isomerases, can be explored using the B6 database tool (Percudani and Peracchi, 2009). These reactions roughly cover the whole spectrum of plant metabolism. In doing so, B6 is required in amino acid synthesis as well as catabolism (Mooney and Hellmann, 2010). This is illustrated by the Arabidopsis mutant *reduced sugar response (rsr4-1)*, harboring a mutated B6 biosynthesis gene (*PDX1*), exhibiting a decreased content of shikimate, altered levels of different amino acids, and higher levels of TCA constituents (malate, citrate and fumarate) (Wagner et al., 2006). Similarly, Arabidopsis mutants for the PLP salvage enzyme PDX3 (involved in B6 vitamer interconversions) contained aberrant amino acid profiles. The initial step in starch breakdown, α-glucan phosphorylase, requires PLP as a cofactor (Mooney and Hellmann, 2010). Furthermore, PLP-dependent enzymes play a role in synthesis of glucosinolates (Mikkelsen et al., 2004). Remarkably, biosynthesis of the plant hormones auxin (Zhao, 2010) and ethylene (Van de Poel and Van Der Straeten, 2014; Vanderstraeten and Van Der Straeten, 2017) as well as ethylene breakdown (Nascimento et al., 2014) involve PLP-requiring enzymes. B6 levels have also been linked to nitrogen metabolism as *pdx3* lines were shown to be ammonium dependent (Colinas et al., 2016). This link is further strengthened by the observation that the ammonium transporter mutant *amt1* has altered B6 levels (Pastor et al., 2014).

On top of its vast influence on plant metabolism via PLP-depending enzymes, B6 plays a crucial role as an antioxidant (Vanderschuren et al., 2013; Fudge et al., 2017). Arabidopsis mutants with a lowered B6 status, exhibit distinct phenotypes including poor seed development, delayed flowering and reduced plant growth, while complete knock-outs are lethal (Vanderschuren et al., 2013). The lowered tolerance of these mutants to salt, high light, ultraviolet light, and oxidative stress illustrate the importance of B6 as a stress protector (Vanderschuren et al., 2013). Upon heat stress, a non-catalytic pyridoxine biosynthesis protein (PDX1.2), ensures sufficient B6 production by aiding its paralogs (PDX1.1 and PDX1.3), resulting in an increase of B6 content (Moccand et al., 2014; Dell’Aglio et al., 2017). Conversely, Arabidopsis lines, engineered for enhanced B6 content, display enhanced tolerance to abiotic stresses (Raschke et al., 2011). Furthermore, these plants exhibit enlarged cells, leading to larger organs. Interestingly, their amino acid and sugar composition is severely altered, reflecting the broad influence of B6 on plant metabolism.

### Pathophysiology and Epidemiology

Vitamin B6, especially PLP, is crucial for correct human functioning, as it is required as a cofactor for around 4% of all enzyme activities (Ueland et al., 2017). Most of these reactions involve amino acid synthesis and catabolism, in which PLP serves as a cofactor in transaminations, aldol cleavages and carboxylations. Furthermore, PLP plays a role in energy metabolism as it is involved in gluconeogenesis and lipid metabolism. Moreover, B6 is necessary in the biosynthesis of heme as well as neurotransmitters (Ueland et al., 2017). In addition, B6 plays an important role as an antioxidant (Justiniano et al., 2017) and is even known to aid in enzyme folding (Cellini et al., 2014).

In parallel with its functions in human metabolism, B6 deficiency is manifested in a broad spectrum of disorders. Most notably, B6 deficiency is known to provoke neurological disorders, such as peripheral neuropathy (Ghavanini and Kimpinski, 2014) and epileptic seizures (Skodda and Muller, 2013). Moreover, B6 deficiency might be linked to anemia, given the ability of B6 intake to cure some cases of the disease (Hisano et al., 2010). Furthermore, B6 deficiency has been associated with cardiovascular diseases, stroke, rheumatoid arthritis, diabetes and different types of cancer including colorectal, lung, breast, and kidney (Ueland et al., 2017).

Although investigation on vitamin B6 deficiency on a global scale is lacking, there is evidence supporting the existence of persistent deficiency in several populations (Fudge et al., 2017). Indeed, studies in the United States and South Korea concluded that around one-in-four people have sub-optimal B6 status (Pfeiffer et al., 2013; Kim and Cho, 2014). Furthermore, half of the elderly in nursing homes in Norway were considered B6 deficient (Kjeldby et al., 2013). The situation in developing countries is estimated to be even worse, given the observation that over half of the population of Uganda and Sudan remain B6 deficient (Fudge et al., 2017). Knowing the detrimental effect this deficiency, remaining undiagnosed, could exert on human health, there is a strong need to supply these people with satisfactory amounts of B6.

Humans, unable to synthesize B6 *de novo*, predominantly depend on their diet for sufficient B6 acquisition, as gut bacteria can be considered as suppliers of marginal amounts of different vitamins (LeBlanc et al., 2013; Fudge et al., 2017). Good sources of dietary B6, besides animal-derived products such as fish and meat, are fresh vegetables including carrots and onions (USDA, 2016; Fudge et al., 2017). However, bioavailability should be considered, given the observations that up to half of the B6 pool could be lost as a result of incomplete digestibility, which is found to be more problematic in plant-based food sources compared to animal products (Roth-Maier et al., 2002). Furthermore, the most consumed staple crops in the world are considered poor sources of dietary B6 (Fudge et al., 2017) (**Table 1**).

### Biofortification

Metabolic engineering approaches rely on the knowledge acquired of the relatively simple plant B6 biosynthesis pathway, mainly involving PDX2 (Tambasco-Studart et al., 2007) and the pyridoxal phosphate synthase protein (PDX1) (Titiz et al., 2006). In a metabolic engineering strategy, overexpression of both *PDX1* and *PDX2* genes yielded up to fourfold increase in B6 levels, while overexpression of the single genes only generated marginal effects (Raschke et al., 2011). Interestingly, enhanced plant biomass in aerial organs with similar overall morphology as well as tolerance to oxidative stress were observed in two-gene engineered plants with increased B6 content. When targeted to roots, the two-gene approach, enabled almost sixfold augmentation of B6 in cassava, without any severe alteration in yield (Li et al., 2015). The success of this two-gene engineering strategy therefore supports assessment in different crops, as well as investigation of possible influences on crop physiology and yield.

So far, analysis of crop germplasm has revealed limited variation (<2-fold) in B6 composition of potato (Mooney et al., 2013) and wheat (Shewry et al., 2011). However, screening of vast accessions of a particular crop could identify interesting lines and thereby also pinpoint novel important QTLs and maybe novel genes influencing B6 homeostasis (Fudge et al., 2017).

## Vitamin B9

**FIGURE 4:**
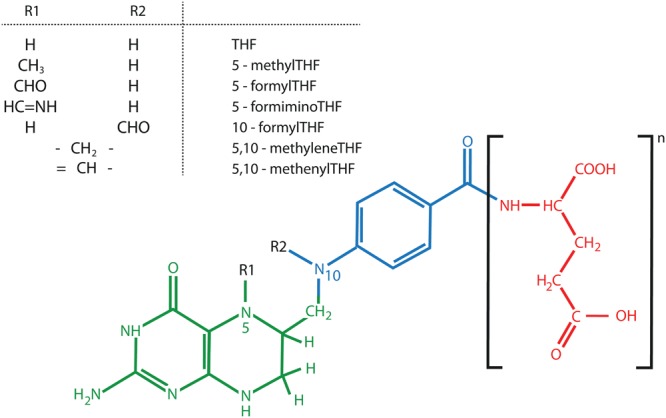
Chemical structure of folates (B9). The three different folate components, pterin (green), para-aminobenzoate (blue), and glutamate tail (red) are indicated. Here, a fully reduced tetrahydrofolate (THF) is presented. Figure adopted from (Strobbe and Van Der Straeten, 2017).

Folate is a collective term for a group of water soluble B9 vitamins. Folates can be considered tri-partite structures, consisting of a pterin ring linked to the *para*-aminobenzoate (*p*-ABA) moiety carrying a γ-linked glutamate tail (Scott et al., 2000; Rebeille et al., 2006) (**Figure 4**). The different folate species, called vitamers, are chemically different on three levels, being the oxidation state, the glutamate tail length and the nature of C1-substituents (Blancquaert et al., 2010; Strobbe and Van Der Straeten, 2017). These properties all exert an influence on folate stability. First, oxidized folates are considered more stable, given the susceptibility of the pterin – *p-*ABA linkage to (photo-) oxidative cleavage (Blancquaert et al., 2010). Tetrahydrofolates (THF), the most reduced folate forms, harboring a fully reduced B-ring in the pterin moiety, are the active cofactors. Conversely, folic acid, containing an aromatic pterin B-ring, is more stable, though exhibiting marginal natural occurrence (Blancquaert et al., 2010; Gorelova et al., 2017b). In this respect, the term ‘folic acid’ is used to indicate the synthetic folate analog. Second, folate entities greatly differ in their glutamate tail length, as they carry one to eight glutamates (Garratt et al., 2005; Strobbe and Van Der Straeten, 2017). Polyglutamylated folates are thought to possess enhanced *in vivo* stability as their polyglutamate tail ensures cellular retention as well as augmented association with folate dependent enzymes (Blancquaert et al., 2014). Third, folates species can differ in their attached C1- units, giving rise to an array of folate entities, affecting their stability and biological role (**Figure 4**).

### Biosynthesis

**FIGURE 5:**
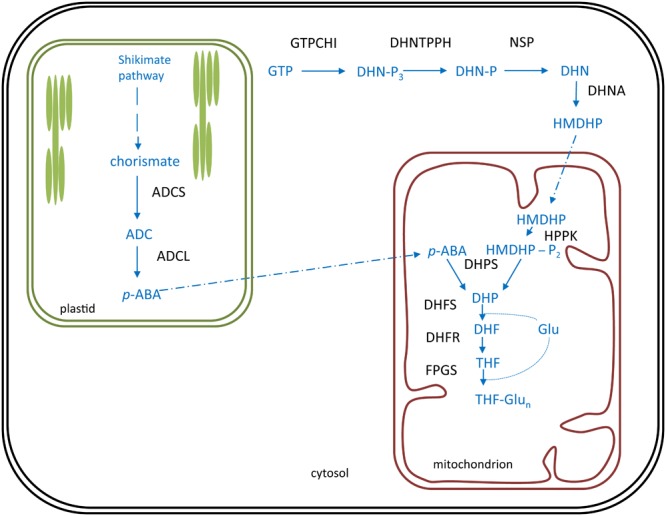
Folate biosynthesis is plants. Folate (vitamin B9) biosynthesis in plants occurs in three subcellular compartments: the cytosol, the plastids (green) and the mitochondrion (red). Biosynthesis pathway is shown in blue, enzymes in black. Polyglutamylated folates are considered the end product of folate biosynthesis. Products: ADC, aminodeoxychorismate; *p*-ABA, *para*-aminobenzoate; DHN-P_3_, dihydroneopterin triphosphate; DHN-P, dihydroneopterin monophosphate; DHN, dihydroneopterin; HMDHP, 6-hydroxymethyldihydropterin; HMDHP-P2, HMDHP pyrophosphate; DHP, dihydropteroate; DHF, dihydrofolate; Glu, glutamate; THF, tetrahydrofolate. Enzymes: ADCS, ADC synthase; ADCL, ADC lyase; GTPCHI, GTP cyclohydrolase I; DHNTPPH, dihydroneopterin triphosphate pyrophophohydrolase; NSP, non-specific phosphatase; DHNA, DHN aldolase; HPPK, HMDHP pyrophosphokinase; DHPS, DHP synthase; DHFS, DHF synthetase; DHFR, DHF reductase; FPGS, folylpolyglutamate synthetase.

In plants, folate biosynthesis is executed in different subcellular localizations (**Figure 5**). The pterin ‘branch’ resides in the cytosol (Strobbe and Van Der Straeten, 2017). Here, the first committed step is executed by GTP cyclohydrolase I (GTPCHI), utilizing GTP as a substrate and yielding 6-hydroxymethyldihydropterin (HMDHP) (Basset et al., 2002). An alleged pterin mitochondrial importer is considered to ensure translocation of HMDHP to the mitochondrion (Hanson and Gregory, 2011; Strobbe and Van Der Straeten, 2017). The plastidial *p*-ABA branch supplies the *p*-ABA moiety of the folate molecule (**Figure 5**). Here, the first committed step is performed by aminodeoxychorismate synthase (ADCS), using chorismate, originating from the shikimate pathway (Herrmann and Weaver, 1999), as a substrate (Sahr et al., 2006). Given the hydrophobic nature of *p*-ABA, it is thought to reach the mitochondria by diffusion through membranes (Hanson and Gregory, 2011; Strobbe and Van Der Straeten, 2017). Upon entering the mitochondria, HMDHP is pyrophosphorylated and coupled with *p*-ABA to form dihydropteroate. These enzymatic reactions are executed by the bifunctional HMDHP pyrophosphokinase/dihydropteroate synthase (HPPK/DHPS) (Gorelova et al., 2017a). Subsequently, dihydropteroate is converted to dihydrofolate (DHF) by the action of dihydrofolate synthetase (DHFS) (Ravanel et al., 2001), followed by a reduction catalyzed by dihydrofolate reductase as part of a bifunctional enzyme dihydrofolate reductase/thymidylate synthase (DHFR-TS) (Gorelova et al., 2017b), yielding THF. Folate biosynthesis is finalized upon polyglutamylation of THF, by the action of folylpolyglutamate synthetase (FPGS) (Ravanel et al., 2001; Mehrshahi et al., 2010).

### Role in Plant Physiology

The chemical structure of folates makes them ideal carriers of C_1_-substituents, conferring a central role in carbon metabolism of nearly all living organisms (Blancquaert et al., 2010), with the exception of some Archaea (Gorelova et al., 2017b). Thereby, folates are both needed for proper anabolism as well as catabolism of cellular compounds. They play an essential role in the synthesis of purines as well as thymidylate and are therefore indispensable in DNA synthesis and growth (Stover, 2004). Furthermore, folates are required in biosynthesis of many plant metabolites including pantothenate (vitamin B5) and formyl methionyl tRNA as well as serine and glycine interconversion and catabolism of histidine (Blancquaert et al., 2010). Furthermore, folates are needed in production of lignin, ensuring cell wall rigidity (Srivastava et al., 2015). In addition, iron-sulfur cluster enzymes depend on folates for their assembly (Waller et al., 2010). Given their role as C1 donors and acceptors, folates play a key role in the methyl cycle (Blancquaert et al., 2010). 5-methyl-THF, is required as a methyl-donor in the conversion of homocysteine to methionine, which is necessary for replenishing of the SAM-pool (Blancquaert et al., 2010). SAM in its turn, functions as methyl storage in supplying this C_1_-unit to a wide range of methyltransferases, including DNA methyltransferases. Therefore, insufficient folate can alter the methyl-cycle homeostasis and evoke epigenetic changes by alteration in the DNA methylation pattern (Zhou et al., 2013). A disequilibrated folate homeostasis greatly influences epigenetic functioning through genome-wide hypomethylation, lowered histone methylation and transposon derepression, as witnessed in Arabidopsis methyleneTHF dehydrogenase/methenylTHF cyclohydrolase (*MTHFD1*) mutants (Groth et al., 2016). Similarly, aberrant functioning of FPGS, the enzyme responsible for extension of the glutamate tail, evoked upregulation of transposable elements (typically repressed by methylation), which could be reverted via administration of 5-methyl-THF (Zhou et al., 2013).

Additional to their requirement in catabolism and anabolism of essential plant metabolites, folates appear to have a profound influence on plant growth and development. In non-photosynthetic plastids, the plastidial pool of folates influences plant energy metabolism by inhibiting starch formation (Hayashi et al., 2017). The mechanism is thought to operate via depletion of the ATP pool -required in starch assembly from sucrose- upon folate shortage, regulated by the folate-dependent DHFR-TS (Hayashi et al., 2017). Remarkably, the interplay of folate and sugar metabolism was shown to modulate auxin signaling, hence controlling plant development (Stokes et al., 2013). Moreover, folates possess the ability to influence seed composition, demonstrated by the high N-content of Arabidopsis plastidial FPGS (*atdfb-3*) loss-of-function mutant seeds (Meng et al., 2014). This reveals an interaction between folate metabolism and N-metabolism in darkness. Folate metabolism was also shown to maintain root development in the indeterminate state, via FPGS functioning (Reyes-Hernandez et al., 2014). Folate synthesis and therefore accumulation is high during germination and in meristematic tissues, coherent with their demand upon cell division and concomitant DNA synthesis (Rebeille et al., 2006). Moreover, folate biosynthesis is stimulated upon light exposure, indicating a higher folate requirement (Rebeille et al., 2006). Indeed, the production of chlorophyll is dependent on folate (Van Wilder et al., 2009). Moreover, folates are able to ensure sufficient NADPH production, thereby controlling cellular redox state by a balanced functioning of *DHFR-TS* genes, needed in detoxification of ROS originating from photosynthesis or photorespiration (Gorelova et al., 2017b). In photorespiration, folate is directly required as a cofactor for the serine hydroxymethyltransferase in the glycine decarboxylase complex (Collakova et al., 2008; Maurino and Peterhansel, 2010). Finally, folate biosynthesis enzymes are known to influence plant stress responses, possibly through generation of folate biosynthesis intermediates (Storozhenko et al., 2007b; Navarrete et al., 2012).

Given the influence of folates on plant development, their homeostasis and accumulation is considered to be tightly regulated, depending on their tissue specific requirement (Rebeille et al., 2006). Indeed, recent insights in folate metabolism of Arabidopsis confirm fine-tuning of folate accumulation by feed-back inhibition of a regulatory DHFR-TS homolog (DHFR-TS3) (Gorelova et al., 2017b). Together, these findings raise caution toward possible implications upon folate biofortification, as an increased folate pool might influence different aspects of plant physiology (Van Wilder et al., 2009).

### Pathophysiology and Epidemiology

Humans lack the ability to synthetize folates *de novo*. However, they possess DHFR and FPGS enzymes, thereby allowing conversion of DHF to THF and polyglutamylated folates, respectively (Masters and Attardi, 1983; Garrow et al., 1992). Hence, humans are almost completely reliant on their diet for adequate folate supply, given that the gut microbiome has a marginal contribution to the folate pool (Camilo et al., 1996; LeBlanc et al., 2013). As the usage of folates as C1 donors and acceptors originated early in evolution, being implemented by prokaryotes and all eukaryotes, their basic functioning in plants is very similar to that in humans. Thus, folates are important in DNA synthesis and in supplying methyl groups to proteins, lipids, and DNA, through their necessity in SAM replenishment (Saini et al., 2016). Similar to plants, changes in folates levels have the potency to change the human epigenome (Bistulfi et al., 2010). Folates are required in methylation of myelin basic protein, which is pivotal for the compaction of myelin around the neuron sheath, thereby ensuring sufficient nerve conduction (Ramaekers and Blau, 2004; Bottiglieri, 2005).

Upon inadequate dietary folate intake, folate status can drop, a condition known as folate deficiency, which has a broad pathophysiology. Folate deficiency results in decreased erythrocyte development, causing megaloblastic anemia (Lanzkowsky, 2016). The elevated levels of homocysteine, resulting from low folate status, can induce vascular diseases, such as coronary artery disease and strokes (Antoniades et al., 2009; Guo et al., 2009; Zeng et al., 2015). The most notable consequence of folate deficiency is its detrimental impact on neurulation. This is revealed by the occurrence of neural tube defects (NTDs) such as *spina bifida*, encephalocele and anencephaly, caused by folate deficiency (Geisel, 2003; Youngblood et al., 2013; Greene and Copp, 2014). Last but not least, different forms of cancer have been linked to inadequate folate status, including colorectal (Feng et al., 2017), prostate (Price et al., 2016), and pancreatic tumors (Yallew et al., 2017).

Folate deficiency is still a global problem, predominantly present in the developing world, yet persisting in many populations of the developed world as well (Blancquaert et al., 2014; Zaganjor et al., 2015). Moreover, even populations blessed by the availability and opportunity of a diverse and folate-rich diet, remain susceptible to deficiency, as illustrated by the low folate status measured in the Swedish population (Eussen et al., 2013; Gylling et al., 2014) and the observed sub-optimal folate levels in 39% of Belgian first trimester pregnancies (Vandevijvere et al., 2012). Worldwide, 300,000 pregnancies are estimated to be affected by NTDs annually, half of which are considered to be caused by insufficient maternal folate status (Flores et al., 2014). China, inhabited by almost 1.4 billion people, recorded a countrywide prevalence of NTDs as high as 0.24% (Blancquaert et al., 2014). More strikingly, Shanxi province, located in Northern China, has amongst the highest incidence rates of NTDs in the world, as high as 1.39% (Li et al., 2006).

Fortunately, noteworthy advances have been made in the fight against folate malnutrition. Educational efforts, advocating a diverse diet containing folate rich foods such as green leafy vegetables and fermented products, is the primary strategy to diminish folate deficiency (Strobbe and Van Der Straeten, 2017). Folic acid, the synthetic form of folate as administered in pills, has been implemented in fortification strategies, which have ensured a significant reduction of neural tube defects (Williams et al., 2015; Wang et al., 2016). Unfortunately, high folic acid intake can also impose unwanted side effects, since excessive accumulation of unmetabolized folic acid has been linked to colorectal cancer and impaired immunity (Cho et al., 2015; Selhub and Rosenberg, 2016). Moreover, both folic acid fortification and supplementation are costly interventions, which are difficult to implement in poor rural regions in need (Blancquaert et al., 2014). Therefore, biofortification, via metabolic engineering or breeding is advised to ensure a stable cost-effective means to fight folate deficiency (De Steur et al., 2012, 2015; Blancquaert et al., 2014; Strobbe and Van Der Straeten, 2017).

### Biofortification

Over the last decades, many successful folate biofortification approaches have been conducted, thereby additionally acquiring new insights in folate metabolism in certain crops and tissues (De Lepeleire et al., 2017; Strobbe and Van Der Straeten, 2017). The most widely attempted folate metabolic engineering approach is the enhancement of GTPCHI activity, proven to be a fruitful strategy in prokaryotes (Sybesma et al., 2003). This approach has been confirmed to be functional in plants by the engineering of *cis*-genic Arabidopsis lines, over-expressing *GTPCHI* (Hossain et al., 2004). This single gene approach, introducing *GTPCHI*, referred to as G-engineering, has been implemented in rice (Storozhenko et al., 2007a), tomato (de la Garza et al., 2004), maize (Naqvi et al., 2009), lettuce (Nunes et al., 2009), potato (Blancquaert et al., 2013a), and Mexican common bean (Ramírez Rivera et al., 2016). The highest fold enhancement, reached in the edible portions of these crops is a ninefold folate increase in lettuce. This could possibly be due to a difference in regulation in leafy tissue. However, single gene approaches have hitherto not resulted in over 10-fold increase in folate content. A bigenic approach was substantially more successful, adding ectopic expression of aminodeoxychorismate synthase (ADCS) (GA-strategy). In tomato (de la Garza et al., 2007) and rice (Storozhenko et al., 2007a) this led to 25- and 100-fold folate enhancement, respectively. Unfortunately, this approach, able to reach the desired levels in tomato and rice, does not promise to be universally applicable, as it only resulted in limited enhancements in Arabidopsis and potato (Blancquaert et al., 2013a). In rice seeds, ADCS has been indicated as the most important limiting factor in folate biosynthesis, additional to GTPCHI (Dong et al., 2014a). Building further on these findings, novel biofortification approaches aimed at further gene stacking, using mitochondrial folate biosynthesis genes (Strobbe and Van Der Straeten, 2017). Indeed, additional introduction of *FPGS* in GA-engineered plants did not only result in elevated folate levels in rice endosperm (100-fold) and potato tubers (12-fold) respectively, but also in enhanced folate stability upon storage (Blancquaert et al., 2015; De Lepeleire et al., 2017). Increasing storage stability has also been addressed by introduction of mammalian folate binding proteins (Blancquaert et al., 2015). This strategy is promising, as it could limit the aforementioned undesired effects of folate increase on plant physiology, via sequestration of the active folate pool. Moreover, recent discovery of plant folate binding proteins creates novel opportunities in folate biofortification via metabolic engineering (Puthusseri et al., 2018).

Breeding endeavors, aimed at acquiring elite crop variants with augmented folate content in the edible portion, though not implemented so far, have shown to be feasible (Andersson et al., 2017; Bouis and Saltzman, 2017). Upon availability of high throughput folate quantification in the food matrix, screening of vast germplasm collections could lead to identification of high folate varieties (De Brouwer et al., 2010; Strobbe and Van Der Straeten, 2017). In this respect, over sevenfold variation in milled rice folate content was described by examination of 78 accessions (Dong et al., 2011). More recently, unpolished brown rice folate content was found to vary up to threefold in 150 examined accessions (Aiyswaraya et al., 2017). Similar screening has been employed in barley (Andersson et al., 2008), red beet (Wang and Goldman, 1996), potato (Goyer and Sweek, 2011; Robinson et al., 2015), tomato (Iniesta et al., 2009), muskmelon (Lester and Crosby, 2002), common bean (Khanal et al., 2011; Jha et al., 2015), lentil (Jha et al., 2015), (chick)pea (Jha et al., 2015), spinach (Shohag et al., 2011), and strawberry (Mezzetti et al., 2016). Furthermore, these variations could be utilized to identify interesting QTLs, underlying folate content, in GWAS (Khanal et al., 2011; Dong et al., 2014b). These techniques, though limited in their potential folate enhancement, are promising, as they might face lower regulatory restrictions, hence allow more rapid implementation in agriculture, reaching the populations in need (Mejia et al., 2017; Potrykus, 2017).

## B-Vitamin Interplay

**FIGURE 6:**
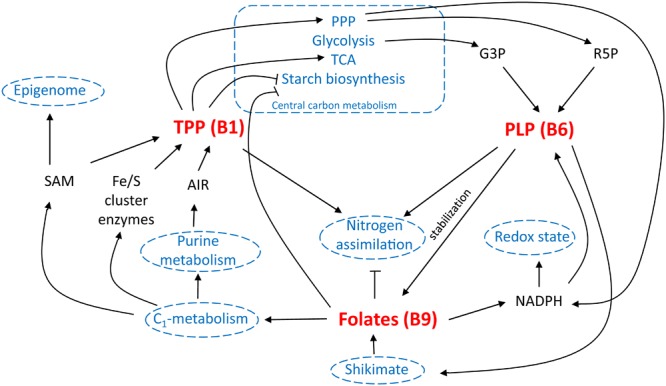
B-vitamin interplay *in planta*. Different interactions of vitamin B1 [via thiamin pyrophosphate (TPP) functioning], B6 [via pyridoxal phosphate (PLP) functioning] and B9 (folates) are schematically displayed. Well-established links are indicated in black, potential interactions are indicated in blue. TCA, tricarboxylic acid cycle; PPP, pentose phosphate pathway; G3P, glyceraldehyde 3-phosphate; R5P, ribose 5′-phosphate; SAM, *S*-adenosylmethionine; AIR, 5-aminoimidazole ribonucleotide; NADPH, nicotinamide adenine dinucleotide phosphate.

Multi-biofortification is considered an important goal in the fight against MNM (Blancquaert et al., 2014; Strobbe and Van Der Straeten, 2017). However, possible effects of altered micronutrient levels upon each other as well as on basic plant growth and development, should be taken into consideration. Examination of the role of B-vitamins in plant metabolism evidently reveals that inducing their accumulation could alter plant physiology. This has been conspicuously observed in metabolic engineering approaches of B1 (Bocobza et al., 2013; Dong et al., 2015) and B6 (Raschke et al., 2011). Furthermore, B9 enhancement, though not depicting any severe effect on plant growth, has shown to alter the rice seed metabolism (Blancquaert et al., 2013b). The influence of B-vitamins on plant metabolism is, however, at least partly intertwined, indicating the importance of detailed investigation of the effect of their combined biofortification (**Figure 6**).

In central energy metabolism, both folate and B1 appear to negatively influence the plant’s ability to accumulate starch (Bocobza et al., 2013; Hayashi et al., 2017). PLP (B6) is also involved in starch breakdown, though there are no indications to suspect increased starch breakdown upon elevation of PLP levels (Zeeman et al., 2004; Mooney and Hellmann, 2010). In the biosynthesis of B6, G3P, an intermediate in central energy metabolism (glycolysis), serves as a substrate (Fudge et al., 2017), the steady state concentration of which might be altered in B1 engineered lines (Bocobza et al., 2013). In altering this central metabolism equilibrium, B1 augmentation might influence the flux through the shikimate pathway (Bocobza et al., 2013), the activity of which is required in the plastidial part of folate biosynthesis (Strobbe and Van Der Straeten, 2017). In this shikimate pathway, PLP (B6) is required as cofactor. Folates are able to generate NADPH (Gorelova et al., 2017b), replenish the SAM pool (Blancquaert et al., 2010), and are needed in the biosynthesis of iron sulfur cluster enzymes (Waller et al., 2010). Interestingly, THIC, pinpointed as the rate limiting step in B1 biosynthesis, contains an iron-sulfur cluster and requires SAM for its catalytic activity (Pourcel et al., 2013). Strongly increased THIC activity would therefore require enhanced SAM turnover (Palmer and Downs, 2013), for which enhanced folate levels might have a beneficial effect. NADPH is on the other hand required for pyridoxal reductase activity in B6 homeostasis. Ribose 5′-phosphate, an important substrate in B6 biosynthesis (Fudge et al., 2017), is a product of the pentose phosphate pathway, the flux of which might be controlled by B1 (Bocobza et al., 2013). Similarly, AIR, an important substrate in B1 biosynthesis (Pourcel et al., 2013), is derived from purine metabolism, the synthesis of which is dependent on folate (Strobbe and Van Der Straeten, 2017). Moreover, B1, B6, and B9 have been linked to nitrogen metabolism. First, thiamin application is known to stimulate nitrogen assimilation (Bahuguna et al., 2012). Second, B6 content was observed to be altered in the ammonium transporter *mutant amt1* (Pastor et al., 2014). Moreover, PLP (B6) salvage mutant *pdx3* is depending on ammonium (Colinas et al., 2016). Third, folate biosynthesis mutants (*atdfb-3*, plastidial FPGS) harbored enhanced nitrogen content of seeds (Meng et al., 2014). Remarkably, given the labile nature of folate, increasing *in planta* stabilization of folates has been the subject of biofortification strategies (Blancquaert et al., 2015). Therefore, enhancing levels of antioxidants, such as B6, has been proposed as an additional biofortification strategy, protecting the folate pool from oxidative cleavage (Blancquaert et al., 2014).

## Final Remarks

Creation and evaluation of multi-biofortified crops would not only offer a sustainable solution to eradicate MNM, but also help to elucidate the interplay of different micronutrients. The availability of novel tools, allowing facilitated cloning of multiple genes paved the way toward such multi-biofortification (Engler et al., 2014). Furthermore, a prerequisite in biofortification strategies is to consider stability upon storage of the crop product, as well as after food processing and bioavailability upon human consumption (Blancquaert et al., 2015; Diaz-Gomez et al., 2017). Different agronomical techniques could be employed, alone or in combination, to augment vitamin content of crops. Metabolic engineering of the complete pathway, or symbiosis with bacteria, might be appropriate ways to tackle vitamin B12 deficiency (DeMell and Holland, 2016). Metabolic engineering strategies could be developed in a precise way, enabling the creation of food crops which harbor an ideal balance of energy supply and micronutrient delivery, while exhibiting marginal effects on plant physiology. These novel crop varieties could, in combination with fortification and dietary interventions eradicate MNM, alleviating a great global burden.

## Author Contributions

All authors listed have made a substantial, direct and intellectual contribution to the work, and approved it for publication.

## Conflict of Interest Statement

The authors declare that the research was conducted in the absence of any commercial or financial relationships that could be construed as a potential conflict of interest.

## Supplementary Material

TABLE S1 An overview of the abbreviations.

## References

[B1] Abdou E. Hazell A. S. (2015). Thiamine deficiency: an update of pathophysiologic mechanisms and future therapeutic considerations. *Neurochem. Res.* 40 353–361. 10.1007/s11064-014-1430-z 25297573

[B2] Adeva-Andany M. M. Lopez-Maside L. Donapetry-Garcia C. Fernandez-Fernandez C. Sixto-Leal C. (2017). Enzymes involved in branched-chain amino acid metabolism in humans. *Amino Acids* 49 1005–1028. 10.1007/s00726-017-2412-7 28324172

[B3] Ahn I. P. Kim S. Lee Y. H. Suh S. C. (2007). Vitamin B_1_-induced priming is dependent on hydrogen peroxide and the *NPR1* gene in *Arabidopsis*. *Plant Physiol.* 143 838–848. 10.1104/pp.106.092627 17158583 PMC1803731

[B4] Ahoua L. Etienne W. Fermon F. Godain G. Brown V. Kadjo K. (2007). Outbreak of beriberi in a prison in Cote d’Ivoire. *Food Nutr. Bull.* 28 283–290. 10.1177/156482650702800304 17974361

[B5] Aiyswaraya S. Saraswathi R. Ramchander S. Jaivel N. Uma D. Sudhakar D. (2017). Evaluation of rice (*Oryza sativa* L.) germplasm for the identification of high folate accession using HPLC. *Int. J. Curr. Microbiol. App. Sci.* 6 5328–5346. 10.20546/ijcmas.2017.611.509

[B6] Ajjawi I. Rodriguez Milla M. A. Cushman J. Shintani D. K. (2007a). Thiamin pyrophosphokinase is required for thiamin cofactor activation in *Arabidopsis*. *Plant Mol. Biol.* 65 151–162. 17611796 10.1007/s11103-007-9205-4

[B7] Ajjawi I. Tsegaye Y. Shintani D. (2007b). Determination of the genetic, molecular, and biochemical basis of the *Arabidopsis thaliana* thiamin auxotroph th1. *Arch. Biochem. Biophys.* 459 107–114. 17174261 10.1016/j.abb.2006.11.011

[B8] Andersson A. A. Lampi A. M. Nystrom L. Piironen V. Li L. Ward J. L. (2008). Phytochemical and dietary fiber components in barley varieties in the HEALTHGRAIN diversity screen. *J. Agric. Food Chem.* 56 9767–9776. 10.1021/jf802037f 18921979

[B9] Andersson M. S. Saltzman A. Virk P. Pfeiffer W. (2017). Progress update: crop development of biofortified staple food crops under HarvestPlus. *Afr. J. Food Agric. Nutr. Dev.* 17 11905–11935. 10.18697/ajfand.78.HarvestPlus05

[B10] Antoniades C. Antonopoulos A. S. Tousoulis D. Marinou K. Stefanadis C. (2009). Homocysteine and coronary atherosclerosis: from folate fortification to the recent clinical trials. *Eur. Heart J.* 30 6–15. 10.1093/eurheartj/ehn515 19029125

[B11] Atta C. A. Fiest K. M. Frolkis A. D. Jette N. Pringsheim T. St Germaine-Smith C. (2016). Global birth prevalence of *spina bifida* by folic acid fortification status: a systematic review and meta-analysis. *Am. J. Public Health* 106 e24–e34. 10.2105/AJPH.2015.302902 26562127 PMC4695937

[B12] Bahuguna R. N. Joshi R. Shukla A. Pandey M. Kumar J. (2012). Thiamine primed defense provides reliable alternative to systemic fungicide carbendazim against sheath blight disease in rice (*Oryza sativa* L.). *Plant Physiol. Biochem.* 57 159–167. 10.1016/j.plaphy.2012.05.003 22705591

[B13] Bailey R. L. West K. P. Black R. E. (2015). The epidemiology of global micronutrient deficiencies. *Ann. Nutr. Metab.* 66 22–33. 10.1159/000371618 26045325

[B14] Banka S. De Goede C. Yue W. W. Morris A. A. Von Bremen B. Chandler K. E. (2014). Expanding the clinical and molecular spectrum of thiamine pyrophosphokinase deficiency: a treatable neurological disorder caused by TPK1 mutations. *Mol. Genet. Metab.* 113 301–306. 10.1016/j.ymgme.2014.09.010 25458521

[B15] Barennes H. Sengkhamyong K. Rene J. P. Phimmasane M. (2015). Beriberi (thiamine deficiency) and high infant mortality in Northern Laos. *PLoS Neglect. Trop. Dis.* 9:e0003581. 10.1371/journal.pntd.0003581 25781926 PMC4363666

[B16] Basset G. Quinlivan E. P. Ziemak M. J. De La Garza R. D. Fischer M. Schiffmann S. (2002). Folate synthesis in plants: the first step of the pterin branch is mediated by a unique bimodular GTP cyclohydrolase I. *Proc. Natl. Acad. Sci. U.S.A.* 99 12489–12494. 10.1073/pnas.192278499 12221287 PMC129472

[B17] Bilski P. Li M. Y. Ehrenshaft M. Daub M. E. Chignell C. F. (2000). Vitamin B-6 (pyridoxine) and its derivatives are efficient singlet oxygen quenchers and potential fungal antioxidants. *Photochem. Photobiol.* 71 129–134. 10.1562/0031-8655(2000)071<0129:SIPVBP>2.0.CO;2 10687384

[B18] Bistulfi G. Vandette E. Matsui S. I. Smiraglia D. J. (2010). Mild folate deficiency induces genetic and epigenetic instability and phenotype changes in prostate cancer cells. *BMC Biol.* 8:6. 10.1186/1741-7007-8-6 20092614 PMC2845099

[B19] Blancquaert D. De Steur H. Gellynck X. Van Der Straeten D. (2014). Present and future of folate biofortification of crop plants. *J. Exp. Bot.* 65 895–906. 10.1093/jxb/ert483 24574483

[B20] Blancquaert D. De Steur H. Gellynck X. Van Der Straeten D. (2017). Metabolic engineering of micronutrients in crop plants. *Ann. N. Y. Acad. Sci.* 1390 59–73. 10.1111/nyas.13274 27801945

[B21] Blancquaert D. Storozhenko S. Loizeau K. De Steur H. De Brouwer V. Viaene J. (2010). Folates and folic acid: from fundamental research toward sustainable health. *Crit. Rev. Plant Sci.* 29 14–35. 10.1080/07352680903436283

[B22] Blancquaert D. Storozhenko S. Van Daele J. Stove C. Visser R. G. F. Lambert W. (2013a). Enhancing pterin and *para*-aminobenzoate content is not sufficient to successfully biofortify potato tubers and *Arabidopsis thaliana* plants with folate. *J. Exp. Bot.* 64 3899–3909. 10.1093/jxb/ert224 23956417

[B23] Blancquaert D. Van Daele J. Storozhenko S. Stove C. Lambert W. Van Der Straeten D. (2013b). Rice folate enhancement through metabolic engineering has an impact on rice seed metabolism, but does not affect the expression of the endogenous folate biosynthesis genes. *Plant Mol. Biol.* 83 329–349. 10.1007/s11103-013-0091-7 23771598

[B24] Blancquaert D. Van Daele J. Strobbe S. Kiekens F. Storozhenko S. De Steur H. (2015). Improving folate (vitamin B-9) stability in biofortified rice through metabolic engineering. *Nat. Biotechnol.* 33 1076–1078. 10.1038/nbt.3358 26389575

[B25] Bocobza S. E. Malitsky S. Araujo W. L. Nunes-Nesi A. Meir S. Shapira M. (2013). Orchestration of thiamin biosynthesis and central metabolism by combined action of the thiamin pyrophosphate riboswitch and the circadian clock in *Arabidopsis*. *Plant Cell* 25 288–307. 10.1105/tpc.112.106385 23341335 PMC3584542

[B26] Bottiglieri T. (2005). Homocysteine and folate metabolism in depression. *Prog. Neuropsychopharmacol. Biol. Psychiatry* 29 1103–1112. 10.1016/j.pnpbp.2005.06.021 16109454

[B27] Boubakri H. Wahab M. A. Chong J. Bertsch C. Mliki A. Soustre-Gacougnolle I. (2012). Thiamine induced resistance to *Plasmopara viticola* in grapevine and elicited host-defense responses, including HR like-cell death. *Plant Physiol. Biochem.* 57 120–133. 10.1016/j.plaphy.2012.05.016 22698755

[B28] Bouis H. E. Saltzman A. (2017). Improving nutrition through biofortification: a review of evidence from HarvestPlus, 2003 through 2016. *Glob. Food Sec.* 12 49–58. 10.1016/j.gfs.2017.01.009 28580239 PMC5439484

[B29] Butterworth R. F. (1993). Pathophysiologic mechanisms responsible for the reversible (thiamine-responsive) and irreversible (thiamine non-responsive) neurological symptoms of Wernicke’s encephalopathy. *Drug Alcohol Rev.* 12 315–322. 10.1080/09595239300185371 16840290

[B30] Cakmak I. Kutman U. (2017). Agronomic biofortification of cereals with zinc: a review. *Eur. J. Soil Sci.* 69 172–180. 10.1111/ejss.12437

[B31] Camaschella C. (2015). Iron-deficiency anemia. *New Engl. J. Med.* 372 1832–1843. 10.1056/NEJMra1401038 25946282

[B32] Camilo E. Zimmerman J. Mason J. B. Golner B. Russell R. Selhub J. (1996). Folate synthesized by bacteria in the human upper small intestine is assimilated by the host. *Gastroenterology* 110 991–998. 10.1053/gast.1996.v110.pm8613033 8613033

[B33] Cellini B. Montioli R. Oppici E. Astegno A. Voltattorni C. B. (2014). The chaperone role of the pyridoxal 5’-phosphate and its implications for rare diseases involving B6-dependent enzymes. *Clin. Biochem.* 47 158–165. 10.1016/j.clinbiochem.2013.11.021 24355692

[B34] Chatterjee A. Li Y. Zhang Y. Grove T. L. Lee M. Krebs C. (2008). Reconstitution of ThiC in thiamine pyrimidine biosynthesis expands the radical SAM superfamily. *Nat. Chem. Biol.* 4 758–765. 10.1038/nchembio.121 18953358 PMC2587053

[B35] Cho E. Zhang X. H. Townsend M. K. Selhub J. Paul L. Rosner B. (2015). Unmetabolized folic acid in prediagnostic plasma and the risk of colorectal cancer. *J. Natl. Cancer Inst.* 107:djv260. 10.1093/jnci/djv260 26376686 PMC4715248

[B36] Clayton P. T. (2006). B-6-responsive disorders: a model of vitamin dependency. *J. Inherit. Metab. Dis.* 29 317–326. 10.1007/s10545-005-0243-2 16763894

[B37] Colinas M. Eisenhut M. Tohge T. Pesquera M. Fernie A. R. Weber A. P. M. (2016). Balancing of B6 vitamers is essential for plant development and metabolism in *Arabidopsis*. *Plant Cell* 28 439–453. 10.1105/tpc.15.01033 26858304 PMC4790880

[B38] Collakova E. Goyer A. Naponelli V. Krassovskaya I. Gregory J. F. Hanson A. D. (2008). *Arabidopsis* 10-formyl tetrahydrofolate deformylases are essential for photorespiration. *Plant Cell* 20 1818–1832. 10.1105/tpc.108.058701 18628352 PMC2518232

[B39] Copenhagen Consensus (2012). *Solutions to Global Challenges.* Available at: http://www.copenhagenconsensus.com/copenhagen-consensus-iii/outcome

[B40] Conrath U. Beckers G. J. M. Flors V. Garcia-Agustin P. Jakab G. Mauch F. (2006). Priming: getting ready for battle. *Mol. Plant Microbe Interact.* 19 1062–1071. 10.1094/MPMI-19-1062 17022170

[B41] De Brouwer V. Storozhenko S. Stove C. P. Van Daele J. Van Der Straeten D. Lambert W. E. (2010). Ultra-performance liquid chromatography–tandem mass spectrometry (UPLC–MS/MS) for the sensitive determination of folates in rice. *J. Chromatogr. B.* 878 509–513. 10.1016/j.jchromb.2009.12.032 20061193

[B42] de la Garza R. D. Quinlivan E. P. Klaus S. M. J. Basset G. J. C. Gregory J. F. Hanson A. D. (2004). Folate biofortification in tomatoes by engineering the pteridine branch of folate synthesis. *Proc. Natl. Acad. Sci. U.S.A.* 101 13720–13725. 10.1073/pnas.0404208101 15365185 PMC518823

[B43] de la Garza R. I. D. Gregory J. F. Hanson A. D. (2007). Folate biofortification of tomato fruit. *Proc. Natl. Acad. Sci. U.S.A.* 104 4218–4222. 10.1073/pnas.0700409104 17360503 PMC1810332

[B44] De Lepeleire J. Strobbe S. Verstraete J. Blancquaert D. Ambach L. Visser R. G. (2017). Folate biofortification of potato by tuber-specific expression of four folate biosynthesis genes. *Mol. Plant* 11 175–188. 10.1016/j.molp.2017.12.008 29277427

[B45] De Steur H. Blancquaert D. Gellynck X. Lambert W. Van Der Straeten D. (2012). Ex-ante evaluation of biotechnology innovations: the case of folate biofortified rice in China. *Curr. Pharm. Biotechnol.* 13 2751–2760. 10.2174/138920112804724873 23072390

[B46] De Steur H. Blancquaert D. Strobbe S. Lambert W. Gellynck X. Van Der Straeten D. (2015). Status and market potential of transgenic biofortified crops. *Nat. Biotechnol.* 33 25–29. 10.1038/nbt.3110 25574631

[B47] De Steur H. Demont M. Gellynck X. Stein A. J. (2017). The social and economic impact of biofortification through genetic modification. *Curr. Opin. Biotechnol* 44 161–168. 10.1016/j.copbio.2017.01.012 28231514

[B48] Dell’Aglio E. Boycheva S. Fitzpatrick T. B. (2017). The pseudoenzyme PDX1.2 sustains vitamin B-6 biosynthesis as a function of heat stress. *Plant Physiol.* 174 2098–2112. 10.1104/pp.17.00531 28550206 PMC5543961

[B49] DeMell A. Holland M. (2016). Enhancement of folate levels in lettuce via a methylotrophic symbiont. *FASEB J.* 30:1143.

[B50] Diaz-Gomez J. Twyman R. M. Zhu C. F. Farre G. Serrano J. C. E. Portero-Otin M. (2017). Biofortification of crops with nutrients: factors affecting utilization and storage. *Curr. Opin. Biotechnol.* 44 115–123. 10.1016/j.copbio.2016.12.002 28068552

[B51] Dong W. Cheng Z. J. Lei C. L. Wang X. L. Wang J. L. Wang J. (2014a). Overexpression of folate biosynthesis genes in rice (*Oryza sativa* L.) and evaluation of their impact on seed folate content. *Plant Foods Hum. Nutr.* 69 379–385. 10.1007/s11130-014-0450-9 25432789

[B52] Dong W. Cheng Z. J. Xu J. L. Zheng T. Q. Wang X. L. Zhang H. Z. (2014b). Identification of QTLs underlying folate content in milled rice. *J. Integr. Agric.* 13 1827–1834. 10.1016/S2095-3119(13)60537-7

[B53] Dong W. Cheng Z. J. Wang X. L. Wang B. Zhang H. Z. Su N. (2011). Determination of folate content in rice germplasm (*Oryza sativa* L.) using tri-enzyme extraction and microbiological assays. *Int. J. Food Sci. Nutr.* 62 537–543. 10.3109/09637486.2011.555476 21438705

[B54] Dong W. Stockwell V. O. Goyer A. (2015). Enhancement of thiamin content in *Arabidopsis thaliana* by metabolic engineering. *Plant Cell Physiol.* 56 2285–2296. 10.1093/pcp/pcv148 26454882

[B55] Dong W. Thomas N. Ronald P. C. Goyer A. (2016). Overexpression of thiamin biosynthesis genes in rice increases leaf and unpolished grain thiamin content but not resistance to *Xanthomonas oryzae* pv. *oryzae*. *Front. Plant Sci.* 7:616. 10.3389/fpls.2016.00616 27242822 PMC4861732

[B56] Elmadfa I. Majchrzak D. Rust P. Genser D. (2001). The thiamine status of adult humans depends on carbohydrate intake. *Int. J. Vitam. Nutr. Res.* 71 217–221. 10.1024/0300-9831.71.4.217 11582856

[B57] Engler C. Youles M. Gruetzner R. Ehnert T. M. Werner S. Jones J. D. G. (2014). A Golden Gate modular cloning toolbox for plants. *ACS Synth. Biol.* 3 839–843. 10.1021/sb4001504 24933124

[B58] Eussen S. J. Nilsen R. M. Midttun O. Hustad S. IJssennagger N. Meyer K. (2013). North-south gradients in plasma concentrations of B-vitamins and other components of one-carbon metabolism in Western Europe: results from the European Prospective Investigation into Cancer and Nutrition (EPIC) Study. *Br. J. Nutr.* 110 363–374. 10.1017/S0007114512004990 23228223

[B59] FAOSTAT (2017). *FAO Statistical Databases FAOSTAT.* Available at: http://www.fao.org/faostat/en/#data

[B60] Feng H. C. Lin J. Y. Hsu S. H. Lan W. Y. Kuo C. S. Tian Y. F. (2017). Low folate metabolic stress reprograms DNA methylation-activated sonic hedgehog signaling to mediate cancer stem cell-like signatures and invasive tumour stage-specific malignancy of human colorectal cancers. *Int. J. Cancer* 141 2537–2550. 10.1002/ijc.31008 28833104

[B61] Flores A. L. Vellozzi C. Valencia D. Sniezek J. (2014). Global burden of neural tube defects, risk factors, and prevention. *Indian J. Community Health* 26 3–5. 26120254 PMC4480200

[B62] Franzese A. Fattorusso V. Mozzillo E. (2017). “Thiamine-responsive megaloblastic anemia syndrome,” in *Diabetes Associated with Single Gene Defects and Chromosomal Abnormalities* eds Barbetti F. Ghizzoni L. Guaraldi F. (Basel: Karger Publishers) 49–54.

[B63] Frelin O. Agrimi G. Laera V. L. Castegna A. Richardson L. G. L. Mullen R. T. (2012). Identification of mitochondrial thiamin diphosphate carriers from *Arabidopsis* and maize. *Funct. Integr. Genomics* 12 317–326. 10.1007/s10142-012-0273-4 22426856

[B64] Fudge J. Mangel N. Gruissem W. Vanderschuren H. Fitzpatrick T. B. (2017). Rationalising vitamin B6 biofortification in crop plants. *Curr. Opin. Biotechnol.* 44 130–137. 10.1016/j.copbio.2016.12.004 28086191

[B65] Gangolf M. Czerniecki J. Radermecker M. Detry O. Nisolle M. Jouan C. (2010). Thiamine status in humans and content of phosphorylated thiamine derivatives in biopsies and cultured cells. *PLoS One* 5:e13616. 10.1371/journal.pone.0013616 21049048 PMC2963613

[B66] Garcia-Casal M. N. Peña-Rosas J. P. Giyose B. (2017). Staple crops biofortified with increased vitamins and minerals: considerations for a public health strategy. *Ann. N. Y. Acad. Sci.* 1390 3–13. 10.1111/nyas.13293 27936288

[B67] Garratt L. C. Ortori C. A. Tucker G. A. Sablitzky F. Bennett M. J. Barrett D. A. (2005). Comprehensive metabolic profiling of mono- and polyglutamated folates and their precursors in plant and animal tissue using liquid chromatography/negative ion electrospray ionisation tandem mass spectrometry. *Rapid Commun. Mass Spectrom.* 19 2390–2398. 10.1002/rcm.2074 16047318

[B68] Garrow T. A. Admon A. Shane B. (1992). Expression cloning of a human cDNA encoding folylpoly(gamma-glutamate) synthetase and determination of its primary structure. *Proc. Natl. Acad. Sci. U.S.A.* 89 9151–9155. 10.1073/pnas.89.19.9151 1409616 PMC50083

[B69] Geisel J. (2003). Folic acid and neural tube defects in pregnancy: a review. *J. Perinat. Neonatal Nurs.* 17 268–279. 10.1097/00005237-200310000-00005 14655787

[B70] Ghavanini A. A. Kimpinski K. (2014). Revisiting the evidence for neuropathy caused by pyridoxine deficiency and excess. *J. Clin. Neuromuscul. Dis.* 16 25–31. 10.1097/CND.0000000000000049 25137514

[B71] Gibson G. E. Blass J. P. Beal M. F. Bunik V. (2005). The alpha-ketoglutarate-dehydrogenase complex: a mediator between mitochondria and oxidative stress in neurodegeneration. *Mol. Neurobiol.* 31 43–63. 10.1385/MN:31:1-3:043 15953811

[B72] Gibson G. E. Hirsch J. A. Cirio R. T. Jordan B. D. Fonzetti P. Elder J. (2013). Abnormal thiamine-dependent processes in Alzheimer’s disease. Lessons from diabetes. *Mol. Cell Neurosci.* 55 17–25. 10.1016/j.mcn.2012.09.001 22982063 PMC3609887

[B73] Godoi P. H. C. Galhardo R. S. Luche D. D. Van Sluys M. A. Menck C. F. M. Oliva G. (2006). Structure of the thiazole biosynthetic enzyme THI1 from *Arabidopsis thaliana*. *J. Biol. Chem.* 281 30957–30966. 10.1074/jbc.M604469200 16912043

[B74] Gorelova V. Ambach L. Rebeille F. Stove C. Van Der Straeten D. (2017a). Folates in plants: research advances and progress in crop biofortification. *Front. Chem.* 5:21. 10.3389/fchem.2017.00021 28424769 PMC5372827

[B75] Gorelova V. De Lepeleire J. Van Daele J. Pluim D. Meï C. Cuypers A. (2017b). Dihydrofolate reductase/thymidylate synthase fine-tunes the folate status and controls redox homeostasis in plants. *Plant Cell* 29 2831–2853. 10.1105/tpc.17.00433 28939595 PMC5728131

[B76] Goyer A. (2010). Thiamine in plants: aspects of its metabolism and functions. *Phytochemistry* 71 1615–1624. 10.1016/j.phytochem.2010.06.022 20655074

[B77] Goyer A. (2017). Thiamin biofortification of crops. *Curr. Opin. Biotechnol.* 44 1–7. 10.1016/j.copbio.2016.09.005 27750185

[B78] Goyer A. Sweek K. (2011). Genetic diversity of thiamin and folate in primitive cultivated and wild potato (*Solanum*) species. *J. Agric. Food Chem.* 59 13072–13080. 10.1021/jf203736e 22088125

[B79] Greene N. D. E. Copp A. J. (2014). Neural tube defects. *Annu. Rev. Neurosci.* 37 221–242. 10.1146/annurev-neuro-062012-170354 25032496 PMC4486472

[B80] Groth M. Moissiard G. Wirtz M. Wang H. F. Garcia-Salinas C. Ramos-Parra P. A. (2016). MTHFD1 controls DNA methylation in *Arabidopsis*. *Nat. Commun.* 7:11640. 10.1038/ncomms11640 27291711 PMC4909953

[B81] Guo H. Y. Chi J. F. Xing Y. B. Wang P. (2009). Influence of folic acid on plasma homocysteine levels & arterial endothelial function in patients with unstable angina. *Indian J. Med. Res.* 129 279–284. 19491420

[B82] Gupta A. (2017). “Epidemiology of nutritional anemia,” in *Nutritional Anemia in Preschool Children* ed. Springer (Singapore: Springer) 7–9. 10.1007/978-981-10-5178-4_2

[B83] Gylling B. Van Guelpen B. Schneede J. Hultdin J. Ueland P. M. Hallmans G. (2014). Low folate levels are associated with reduced risk of colorectal cancer in a population with low folate status. *Cancer Epidemiol. Biomarkers Prev.* 23 2136–2144. 10.1158/1055-9965.EPI-13-1352 25063522

[B84] Haddad L. Hawkes C. Webb P. Thomas S. Beddington J. Waage J. (2016). A new global research agenda for food. *Nature* 540 30–32. 10.1038/540030a 27905456

[B85] Hanson A. D. Gregory J. F. (2011). Folate biosynthesis, turnover, and transport in plants. *Annu. Rev. Plant Biol.* 62 105–125. 10.1146/annurev-arplant-042110-103819 21275646

[B86] Harel Y. Zuk L. Guindy M. Nakar O. Lotan D. Fattal-Valevski A. (2017). The effect of subclinical infantile thiamine deficiency on motor function in preschool children. *Matern. Child Nutr.* 13:e12397. 10.1111/mcn.12397 28133900 PMC6866041

[B87] Harper C. (2006). Thiamine (vitamin B1) deficiency and associated brain damage is still common throughout the world and prevention is simple and safe! *Eur. J. Neurol.* 13 1078–1082. 10.1111/j.1468-1331.2006.01530.x 16987159

[B88] Hayashi M. Tanaka M. Yamamoto S. Nakagawa T. Kanai M. Anegawa A. (2017). Plastidial folate prevents starch biosynthesis triggered by sugar influx into non-photosynthetic plastids of *Arabidopsis*. *Plant Cell Physiol.* 58 1328–1338. 10.1093/pcp/pcx076 28586467 PMC5921527

[B89] Hellmann H. Mooney S. (2010). Vitamin B6: a molecule for human health? *Molecules* 15 442–459. 10.3390/molecules15010442 20110903 PMC6257116

[B90] Herrero S. Gonzalez E. Gillikin J. W. Velez H. Daub M. E. (2011). Identification and characterization of a pyridoxal reductase involved in the vitamin B6 salvage pathway in *Arabidopsis*. *Plant Mol. Biol.* 76 157–169. 10.1007/s11103-011-9777-x 21533842

[B91] Herrmann K. M. Weaver L. M. (1999). The shikimate pathway. *Annu. Rev. Plant Biol.* 50 473–503. 10.1146/annurev.arplant.50.1.473 15012217

[B92] Hisano M. Suzuki R. Sago H. Murashima A. Yamaguchi K. (2010). Vitamin B6 deficiency and anemia in pregnancy. *Eur. J. Clin. Nutr.* 64 221–223. 10.1038/ejcn.2009.125 19920848

[B93] Hoffman R. (2016). Thiamine deficiency in the Western diet and dementia risk. *Br. J. Nutr.* 116 188–189. 10.1017/S000711451600177X 27170224

[B94] Hossain T. Rosenberg I. Selhub J. Kishore G. Beachy R. Schubert K. (2004). Enhancement of folates in plants through metabolic engineering. *Proc. Natl. Acad. Sci. U.S.A.* 101 5158–5163. 10.1073/pnas.0401342101 15044686 PMC387390

[B95] Hsieh W. Y. Liao J. C. Wang H. T. Hung T. H. Tseng C. C. Chung T. Y. (2017). The *Arabidopsis* thiamin-deficient mutant *pale green1* lacks thiamin monophosphate phosphatase of the vitamin B1 biosynthesis pathway. *Plant J.* 91 145–157. 10.1111/tpj.13552 28346710

[B96] Iniesta M. D. Perez-Conesa D. Garcia-Alonso J. Ros G. Periago M. J. (2009). Folate content in tomato (*Lycopersicon esculentum*). Influence of cultivar, ripeness, year of harvest, and pasteurization and storage temperatures. *J. Agric. Food Chem.* 57 4739–4745. 10.1021/jf900363r 19449809

[B97] Ito J. Batth T. S. Petzold C. J. Redding-Johanson A. M. Mukhopadhyay A. Verboom R. (2011). Analysis of the *Arabidopsis* cytosolic proteome highlights subcellular partitioning of central plant metabolism. *J. Proteome Res.* 10 1571–1582. 10.1021/pr1009433 21166475

[B98] Jha A. B. Ashokkumar K. Diapari M. Ambrose S. J. Zhang H. X. Tar’an B. (2015). Genetic diversity of folate profiles in seeds of common bean, lentil, chickpea and pea. *J. Food Compost. Anal.* 42 134–140. 10.1016/j.jfca.2015.03.006

[B99] Jung Y. C. Chanraud S. Sullivan E. V. (2012). Neuroimaging of Wernicke’s encephalopathy and Korsakoff’s syndrome. *Neuropsychol. Rev.* 22 170–180. 10.1007/s11065-012-9203-4 22577003 PMC4728174

[B100] Justiniano R. Williams J. D. Perer J. Hua A. Lesson J. Park S. L. (2017). The B-6-vitamer pyridoxal is a sensitizer of UVA-induced genotoxic stress in human primary keratinocytes and reconstructed epidermis. *Photochem. Photobiol.* 93 990–998. 10.1111/php.12720 28083878 PMC5500433

[B101] Kamarudin A. N. Lai K. S. Lamasudin D. U. Idris A. S. Yusof Z. N. B. (2017). Enhancement of thiamine biosynthesis in oil palm seedlings by colonization of endophytic fungus *Hendersonia toruloidea*. *Front. Plant Sci.* 8:1799. 10.3389/fpls.2017.01799 29089959 PMC5651052

[B102] Kaya C. Ashraf M. Sonmez O. Tuna A. L. Polat T. Aydemir S. (2015). Exogenous application of thiamin promotes growth and antioxidative defense system at initial phases of development in salt-stressed plants of two maize cultivars differing in salinity tolerance. *Acta Physiol. Plant.* 37 1–12. 10.1007/s11738-014-1741-3

[B103] Kennedy G. Burlingame B. (2003). Analysis of food composition data on rice from a plant genetic resources perspective. *Food Chem.* 80 589–596. 10.1016/S0308-8146(02)00507-1

[B104] Khanal S. Shi C. Xue J. Shi J. Rajcan I. Pauls K. P. (2011). Quantitative trait loci analysis of folate content in common beans (*Phaseolus vulgaris* L.). *Can. J. Plant Sci.* 91 375–376.

[B105] Kim Y. N. Cho Y. O. (2014). Evaluation of vitamin B-6 intake and status of 20-to 64-year-old Koreans. *Nutr. Res. Pract.* 8 688–694. 10.4162/nrp.2014.8.6.688 25489409 PMC4252529

[B106] Kjeldby I. K. Fosnes G. S. Ligaarden S. C. Farup P. G. (2013). Vitamin B6 deficiency and diseases in elderly people–a study in nursing homes. *BMC Geriatr.* 13:13. 10.1186/1471-2318-13-13 23394203 PMC3579689

[B107] Kong D. Y. Zhu Y. X. Wu H. L. Cheng X. D. Liang H. Ling H. Q. (2008). AtTHIC, a gene involved in thiamine biosynthesis in *Arabidopsis thaliana*. *Cell Res.* 18 566–576. 10.1038/cr.2008.35 18332905

[B108] Lanzkowsky P. (2016). “Megaloblastic anemia,” in *Lanzkowsky’s Manual of Pediatric Hematology and Oncology* 6th Edn eds Lanzkowsky P. Lipton J. M. Fish J. D. (Cambridge, MA: Academic Press) 84–101. 10.1016/B978-0-12-801368-7.00007-7

[B109] LeBlanc J. G. Milani C. De Giori G. S. Sesma F. Van Sinderen D. Ventura M. (2013). Bacteria as vitamin suppliers to their host: a gut microbiota perspective. *Curr. Opin. Biotechnol.* 24 160–168. 10.1016/j.copbio.2012.08.005 22940212

[B110] Lee D. C. Chu J. Satz W. Silbergleit R. (2000). Low plasma thiamine levels in elder patients admitted through the emergency department. *Acad. Emerg. Med.* 7 1156–1159. 10.1111/j.1553-2712.2000.tb01268.x 11015250

[B111] Lester G. E. Crosby K. M. (2002). Ascorbic acid, folic acid, and potassium content in postharvest green-flesh honeydew muskmelons: influence of cultivar, fruit size, soil type, and year. *J. Am. Soc. Hortic. Sci.* 127 843–847.

[B112] Li C. L. Wang M. Wu X. M. Chen D. H. Lv H. J. Shen J. L. (2016). THI1, a thiamine thiazole synthase, interacts with Ca2+-dependent protein kinase CPK33 and modulates the S-type anion channels and stomatal closure in *Arabidopsis*. *Plant Physiol.* 170 1090–1104. 10.1104/pp.15.01649 26662273 PMC4734576

[B113] Li J. Liu J. Zhang P. Wan Y. Xia X. Zhang Y. (2017). Genome-wide association mapping of vitamins B1 and B2 in common wheat. *Crop J.* (in press) 10.1016/j.cj.2017.08.002

[B114] Li K. T. Moulin M. Mangel N. Albersen M. Verhoeven-Duif N. M. Ma Q. X. (2015). Increased bioavailable vitamin B-6 in field-grown transgenic cassava for dietary sufficiency. *Nat. Biotechnol.* 33 1029–1032. 10.1038/nbt.3318 26448082

[B115] Li Z. W. Ren A. G. Zhang L. Ye R. W. Li S. Zheng J. C. (2006). Extremely high prevalence of neural tube defects in a 4-county area in Shanxi Province, China. *Birth Defects Res.* 76 237–240. 10.1002/bdra.20248 16575897

[B116] Long S. P. Marshall-Colon A. Zhu X. G. (2015). Meeting the global food demand of the future by engineering crop photosynthesis and yield potential. *Cell* 161 56–66. 10.1016/j.cell.2015.03.019 25815985

[B117] Lonsdale D. (2006). Review of the biochemistry, metabolism and clinical benefits of thiamin(e) and its derivatives. *Evid. Based Complement. Alternat. Med.* 3 49–59. 10.1093/ecam/nek009 16550223 PMC1375232

[B118] Lonsdale D. (2015). Sudden infant death syndrome and abnormal metabolism of thiamin. *Med. Hypotheses* 85 922–926. 10.1016/j.mehy.2015.09.009 26455416

[B119] Luxemburger C. White N. J. Ter Kuile F. Singh H. M. Allier-Frachon I. Ohn M. (2003). Beri-beri: the major cause of infant mortality in Karen refugees. *Trans. R. Soc. Trop. Med. Hyg.* 97 251–255. 10.1016/S0035-9203(03)90134-9 14584386

[B120] Mangel N. Fudge J. B. Fitzpatrick T. B. Gruissem W. Vanderschuren H. (2017). Vitamin B-1 diversity and characterization of biosynthesis genes in cassava. *J. Exp. Bot.* 68 3351–3363. 10.1093/jxb/erx196 28859374 PMC5853225

[B121] Martin C. Li J. (2017). Medicine is not health care, food is health care: plant metabolic engineering, diet and human health. *New Phytol.* 216 699–719. 10.1111/nph.14730 28796289

[B122] Masters J. N. Attardi G. (1983). The nucleotide sequence of the cDNA coding for the human dihydrofolic acid reductase. *Gene* 21 59–63. 10.1016/0378-1119(83)90147-6 6687716

[B123] Maurino V. G. Peterhansel C. (2010). Photorespiration: current status and approaches for metabolic engineering. *Curr. Opin. Plant Biol.* 13 249–256. 10.1016/j.pbi.2010.01.006 20185358

[B124] Mehrshahi P. Gonzalez-Jorge S. Akhtar T. A. Ward J. L. Santoyo-Castelazo A. Marcus S. E. (2010). Functional analysis of folate polyglutamylation and its essential role in plant metabolism and development. *Plant J.* 64 267–279. 10.1111/j.1365-313X.2010.04336.x 21070407

[B125] Mejia L. A. Dary O. Boukerdenna H. (2017). Global regulatory framework for production and marketing of crops biofortified with vitamins and minerals. *Ann. N. Y. Acad. Sci.* 1390 47–58. 10.1111/nyas.13275 27801985

[B126] Meng H. Y. Jiang L. Xu B. S. Guo W. Z. Li J. L. Zhu X. Q. (2014). *Arabidopsis* plastidial folylpolyglutamate synthetase is required for seed reserve accumulation and seedling establishment in darkness. *PLoS One* 9:e101905. 10.1371/journal.pone.0101905 25000295 PMC4084893

[B127] Mezzetti B. Balducci F. Capocasa F. Zhong C. F. Cappelletti R. Di Vittori L. (2016). Breeding strawberry for higher phytochemicals content and claim it: is it possible? *Intern. J. Fruit Sci.* 16 194–206. 10.1080/15538362.2016.1250695

[B128] Mikkelsen M. D. Naur P. Halkier B. A. (2004). *Arabidopsis* mutants in the C-S lyase of glucosinolate biosynthesis establish a critical role for indole-3-acetaldoxime in auxin homeostasis. *Plant J.* 37 770–777. 10.1111/j.1365-313X.2004.02002.x 14871316

[B129] Mimura M. Zallot R. Niehaus T. D. Hasnain G. Gidda S. K. Nguyen T. N. D. (2016). *Arabidopsis* TH2 encodes the orphan enzyme thiamin monophosphate phosphatase. *Plant Cell* 28 2683–2696. 10.1105/tpc.16.00600 27677881 PMC5134987

[B130] Moccand C. Boycheva S. Surriabre P. Tambasco-Studart M. Raschke M. Kaufmann M. (2014). The pseudoenzyme PDX1.2 boosts vitamin B-6 biosynthesis under heat and oxidative stress in *Arabidopsis*. *J. Biol. Chem.* 289 8203–8216. 10.1074/jbc.M113.540526 24505140 PMC3961649

[B131] Moll R. Davis B. (2017). Iron, vitamin B-12 and folate. *Medicine* 45 198–203. 10.1016/j.mpmed.2017.01.007

[B132] Mooney S. Chen L. Y. Kuhn C. Navarre R. Knowles N. R. Hellmann H. (2013). Genotype-specific changes in vitamin B_6_ content and the PDX family in potato. *Biomed. Res. Int.* 2013:389723. 10.1155/2013/389723 23971030 PMC3732595

[B133] Mooney S. Hellmann H. (2010). Vitamin B6: killing two birds with one stone? *Phytochemistry* 71 495–501. 10.1016/j.phytochem.2009.12.015 20089286

[B134] Naqvi S. Zhu C. F. Farre G. Ramessar K. Bassie L. Breitenbach J. (2009). Transgenic multivitamin corn through biofortification of endosperm with three vitamins representing three distinct metabolic pathways. *Proc. Natl. Acad. Sci. U.S.A.* 106 7762–7767. 10.1073/pnas.0901412106 19416835 PMC2683132

[B135] Nascimento F. X. Rossi M. J. Soares C. R. F. S. Mcconkey B. J. Glick B. R. (2014). New insights into 1-aminocyclopropane-1-carboxylate (ACC) deaminase phylogeny, evolution and ecological significance. *PLoS One* 9:e99168. 10.1371/journal.pone.0099168 24905353 PMC4048297

[B136] Navarrete O. Van Daele J. Stove C. Lambert W. Van Der Straeten D. Storozhenko S. (2012). A folate independent role for cytosolic HPPK/DHPS upon stress in *Arabidopsis thaliana*. *Phytochemistry* 73 23–33. 10.1016/j.phytochem.2011.09.008 21996493

[B137] Nunes A. C. S. Kalkmann D. C. Aragao F. J. L. (2009). Folate biofortification of lettuce by expression of a codon optimized chicken GTP cyclohydrolase I gene. *Transgenic Res.* 18 661–667. 10.1007/s11248-009-9256-1 19322672

[B138] Palmer L. D. Downs D. M. (2013). The Thiamine biosynthetic enzyme ThiC catalyzes multiple turnovers and is inhibited by S-adenosylmethionine (AdoMet) metabolites. *J. Biol. Chem.* 288 30693–30699. 10.1074/jbc.M113.500280 24014032 PMC3798539

[B139] Pasricha S. R. Low M. Thompson J. Farrell A. De-Regil L. M. (2014). Iron supplementation benefits physical performance in women of reproductive age: a systematic review and meta-analysis. *J. Nutr.* 144 906–914. 10.3945/jn.113.189589 24717371

[B140] Pastor V. Gamir J. Camanes G. Cerezo M. Sanchez-Bel P. Flors V. (2014). Disruption of the ammonium transporter AMT1.1 alters basal defenses generating resistance against *Pseudomonas syringae* and *Plectosphaerella cucumerina*. *Front. Plant Sci.* 5:231. 10.3389/fpls.2014.00231 24910636 PMC4038795

[B141] Percudani R. Peracchi A. (2009). The B6 database: a tool for the description and classification of vitamin B6-dependent enzymatic activities and of the corresponding protein families. *BMC Bioinformatics* 10:273. 10.1186/1471-2105-10-273 19723314 PMC2748086

[B142] Pfeiffer C. M. Sternberg M. R. Schleicher R. L. Haynes B. M. H. Rybak M. E. Pirkle J. L. (2013). The CDC’s second national report on biochemical indicators of diet and nutrition in the U.S. population is a valuable tool for researchers and policy makers. *J. Nutr.* 143 938S–947S. 10.3945/jn.112.172858 23596164 PMC4822995

[B143] Potrykus I. (2017). The GMO-crop potential for more, and more nutritious food is blocked by unjustified regulation. *J. Innov. Knowl.* 2 90–96. 10.1016/j.jik.2017.03.003

[B144] Pourcel L. Moulin M. Fitzpatrick T. B. (2013). Examining strategies to facilitate vitamin B1 biofortification of plants by genetic engineering. *Front. Plant Sci.* 4:160. 10.3389/fpls.2013.00160 23755056 PMC3665906

[B145] Price A. J. Travis R. C. Appleby P. N. Albanes D. Gurrea A. B. Bjorge T. (2016). Circulating folate and vitamin B-12 and risk of prostate cancer: a collaborative analysis of individual participant data from six cohorts including 6875 cases and 8104 controls. *Eur. Urol.* 70 941–951. 10.1016/j.eururo.2016.03.029 27061263 PMC5094800

[B146] Puthusseri B. Divya P. Veeresh L. Kumar G. Neelwarne B. (2018). Evaluation of folate-binding proteins and stability of folates in plant foliages. *Food Chem.* 242 555–559. 10.1016/j.foodchem.2017.09.049 29037729

[B147] Ramaekers V. T. Blau N. (2004). Cerebral folate deficiency. *Dev. Med. Child Neurol.* 46 843–851. 10.1111/j.1469-8749.2004.tb00451.x 15581159

[B148] Ramírez Rivera N. G. García-Salinas C. Aragão F. J. Díaz De La Garza R. I. (2016). Metabolic engineering of folate and its precursors in Mexican common bean (*Phaseolus vulgaris* L.). *Plant Biotechnol. J.* 14 2021–2032. 10.1111/pbi.12561 26997331 PMC5043471

[B149] Rapala-Kozik M. Wolak N. Kujda M. Banas A. K. (2012). The upregulation of thiamin (vitamin B_1_) biosynthesis in *Arabidopsis thaliana* seedlings under salt and osmotic stress conditions is mediated by abscisic acid at the early stages of this stress response. *BMC Plant Biol.* 12:2. 10.1186/1471-2229-12-2 22214485 PMC3261115

[B150] Raschke M. Boycheva S. Crevecoeur M. Nunes-Nesi A. Witt S. Fernie A. R. (2011). Enhanced levels of vitamin B-6 increase aerial organ size and positively affect stress tolerance in *Arabidopsis*. *Plant J.* 66 414–432. 10.1111/j.1365-313X.2011.04499.x 21241390

[B151] Raschke M. Burkle L. Muller N. Nunes-Nesi A. Fernie A. R. Arigoni D. (2007). Vitamin B1 biosynthesis in plants requires the essential iron-sulfur cluster protein, THIC. *Proc. Natl. Acad. Sci. U.S.A.* 104 19637–19642. 10.1073/pnas.0709597104 18048325 PMC2148341

[B152] Rautiainen S. Manson J. E. Lichtenstein A. H. Sesso H. D. (2016). Dietary supplements and disease prevention - a global overview. *Nat. Rev. Endocrinol.* 12 407–420. 10.1038/nrendo.2016.54 27150288

[B153] Ravanel S. Cherest H. Jabrin S. Grunwald D. Surdin-Kerjan Y. Douce R. (2001). Tetrahydrofolate biosynthesis in plants: molecular and functional characterization of dihydrofolate synthetase and three isoforms of folylpolyglutamate synthetase in *Arabidopsis thaliana*. *Proc. Natl. Acad. Sci. U.S.A.* 98 15360–15365. 10.1073/pnas.261585098 11752472 PMC65034

[B154] Rebeille F. Ravanel S. Jabrin S. Douce R. Storozhenko S. Van Der Straeten D. (2006). Folates in plants: biosynthesis, distribution, and enhancement. *Physiol. Plant.* 126 330–342. 10.1111/j.1399-3054.2006.00587.x

[B155] Reinbott A. Schelling A. Kuchenbecker J. Jeremias T. Russell I. Kevanna O. (2016). Nutrition education linked to agricultural interventions improved child dietary diversity in rural Cambodia. *Br. J. Nutr.* 116 1457–1468. 10.1017/S0007114516003433 27702425 PMC5082286

[B156] Reyes-Hernandez B. J. Srivastava A. C. Ugartechea-Chirino Y. Shishkova S. Ramos-Parra P. A. Lira-Ruan V. (2014). The root indeterminacy-to-determinacy developmental switch is operated through a folate-dependent pathway in *Arabidopsis thaliana*. *New Phytol.* 202 1223–1236. 10.1111/nph.12757 24635769

[B157] Robinson B. R. Sathuvalli V. Bamberg J. Goyer A. (2015). Exploring folate diversity in wild and primitive potatoes for modern crop improvement. *Genes* 6 1300–1314. 10.3390/genes6041300 26670256 PMC4690042

[B158] Roman-Campos D. Cruz J. S. (2014). Current aspects of thiamine deficiency on heart function. *Life Sci.* 98 1–5. 10.1016/j.lfs.2013.12.029 24398040

[B159] Roth-Maier D. A. Kettler S. I. Kirchgessner M. (2002). Availability of vitamin B(6) from different food sources. *Int. J. Food Sci. Nutr.* 53 171–179. 10.1080/09637480220132184 11939111

[B160] Ruel-Bergeron J. C. Stevens G. A. Sugimoto J. D. Roos F. F. Ezzati M. Black R. E. (2015). Global update and trends of hidden hunger, 1995-2011: the hidden hunger index. *PLoS One* 10:e0143497. 10.1371/journal.pone.0143497 26673631 PMC4684416

[B161] Sahr T. Ravanel S. Basset G. Nichols B. P. Hanson A. D. Rebeille F. (2006). Folate synthesis in plants: purification, kinetic properties, and inhibition of aminodeoxychorismate synthase. *Biochem. J.* 396 157–162. 10.1042/BJ20051851 16466344 PMC1449997

[B162] Saini R. K. Nile S. H. Keum Y.-S. (2016). Folates: chemistry, analysis, occurrence, biofortification and bioavailability. *Food Res. Int.* 89 1–13. 10.1016/j.foodres.2016.07.013 28460896

[B163] Sandjaja S. Jus’at I. Jahari A. B. Ifrad Htet M. K. Tilden R. L. (2015). Vitamin A-fortified cooking oil reduces vitamin A deficiency in infants, young children and women: results from a programme evaluation in Indonesia. *Public Health Nutr.* 18 2511–2522. 10.1017/S136898001400322X 25591926 PMC10277201

[B164] Sang Y. Y. Barbosa J. M. Wu H. Z. Locy R. D. Singh N. K. (2007). Identification of a pyridoxine (pyridoxamine) 5’-phosphate oxidase from *Arabidopsis thaliana*. *FEBS Lett.* 581 344–348. 10.1016/j.febslet.2006.12.028 17224143

[B165] Scott J. Rebeille F. Fletcher J. (2000). Folic acid and folates: the feasibility for nutritional enhancement in plant foods. *J. Sci. Food Agric.* 80 795–824. 10.1002/(SICI)1097-0010(20000515)80:7<795::AID-JSFA599>3.0.CO;2-K

[B166] Scott S. P. Chen-Edinboro L. P. Caulfield L. E. Murray-Kolb L. E. (2014). The impact of anemia on child mortality: an updated review. *Nutrients* 6 5915–5932. 10.3390/nu6125915 25533005 PMC4277007

[B167] Selhub J. Rosenberg I. H. (2016). Excessive folic acid intake and relation to adverse health outcome. *Biochimie* 126 71–78. 10.1016/j.biochi.2016.04.010 27131640

[B168] Semba R. D. Muhilal M. P. H. West K. P. Winget M. Natadisastra G. Scott A. (1992). Impact of vitamin-A supplementation on hematological indicators of iron-metabolism and protein status in children. *Nutr. Res.* 12 469–478. 10.1016/S0271-5317(05)80017-X

[B169] Shewry P. R. Van Schaik F. Ravel C. Charmet G. Mariann R. Zoltan B. (2011). Genotype and environment effects on the contents of vitamins B1, B2, B3, and B6 in wheat grain. *J. Agric. Food Chem.* 59 10564–10571. 10.1021/jf202762b 21863876

[B170] Shi H. Z. Xiong L. M. Stevenson B. Lu T. G. Zhu J. K. (2002). The *Arabidopsis* salt overly sensitive 4 mutants uncover a critical role for vitamin B6 in plant salt tolerance. *Plant Cell* 14 575–588. 10.1105/tpc.010417 11910005 PMC150580

[B171] Shohag M. J. I. Wei Y. Y. Yu N. Zhang J. Wang K. Patring J. (2011). Natural variation of folate content and composition in spinach (*Spinacia oleracea*) germplasm. *J. Agric. Food Chem.* 59 12520–12526. 10.1021/jf203442h 22004472

[B172] Skodda S. Muller T. (2013). Refractory epileptic seizures due to vitamin B6 deficiency in a patient with Parkinson’s disease under duodopa (R) therapy. *J. Neural Transm.* 120 315–318. 10.1007/s00702-012-0856-1 22798026

[B173] Srivastava A. C. Chen F. Ray T. Pattathil S. Pena M. J. Avci U. (2015). Loss of function of folylpolyglutamate synthetase 1 reduces lignin content and improves cell wall digestibility in *Arabidopsis*. *Biotechnol. Biofuels* 8:224. 10.1186/s13068-015-0403-z 26697113 PMC4687376

[B174] Stokes M. E. Chattopadhyay A. Wilkins O. Nambara E. Campbell M. M. (2013). Interplay between sucrose and folate modulates auxin signaling in *Arabidopsis*. *Plant Physiol.* 162 1552–1565. 10.1104/pp.113.215095 23690535 PMC3707552

[B175] Storozhenko S. De Brouwer V. Volckaert M. Navarrete O. Blancquaert D. Zhang G. F. (2007a). Folate fortification of rice by metabolic engineering. *Nat. Biotechnol.* 25 1277–1279. 17934451 10.1038/nbt1351

[B176] Storozhenko S. Navarrete O. Ravanel S. De Brouwer V. Chaerle P. Zhang G. F. (2007b). Cytosolic hydroxymethyldihydropterin pyrophosphokinase/dihydropteroate synthase from *Arabidopsis thaliana*: a specific role in early development and stress response. *J. Biol. Chem.* 282 10749–10761. 17289662 10.1074/jbc.M701158200

[B177] Stover P. J. (2004). Physiology of folate and vitamin B-12 in health and disease. *Nutr. Rev.* 62 S3–S12. 15298442 10.1111/j.1753-4887.2004.tb00070.x

[B178] Strobbe S. Van Der Straeten D. (2017). Folate biofortification in food crops. *Curr. Opin. Biotechnol.* 44 202–211. 10.1016/j.copbio.2016.12.003 28329726

[B179] Sybesma W. Starrenburg M. Kleerebezem M. Mierau I. De Vos W. M. Hugenholtz J. R. (2003). Increased production of folate by metabolic engineering of *Lactococcus lactis*. *Appl. Environ. Microbiol.* 69 3069–3076. 10.1128/AEM.69.6.3069-3076.2003 12788700 PMC161528

[B180] Tambasco-Studart M. Tews I. Amrhein N. Fitzpatrick T. B. (2007). Functional analysis of PDX2 from *Arabidopsis*, a glutaminase involved in vitamin B6 biosynthesis. *Plant Physiol.* 144 915–925. 10.1104/pp.107.096784 17468224 PMC1914173

[B181] Tambasco-Studart M. Titiz O. Raschle T. Forster G. Amrhein N. Fitzpatrick T. B. (2005). Vitamin B6 biosynthesis in higher plants. *Proc. Natl. Acad. Sci. U.S.A.* 102 13687–13692. 10.1073/pnas.0506228102 16157873 PMC1224648

[B182] Titiz O. Tambasco-Studart M. Warzych E. Apel K. Amrhein N. Laloi C. (2006). PDX1 is essential for vitamin B6 biosynthesis, development and stress tolerance in *Arabidopsis*. *Plant J.* 48 933–946. 10.1111/j.1365-313X.2006.02928.x 17227548

[B183] Trumbo P. Yates A. A. Schlicker S. Poos M. (2001). Dietary reference intakes: vitamin A, vitamin K, arsenic, boron, chromium, copper, iodine, iron, manganese, molybdenum, nickel, silicon, vanadium, and zinc. *J. Am. Diet. Assoc.* 101 294–301. 10.1016/S0002-8223(01)00078-5 11269606

[B184] Tunc-Ozdemir M. Miller G. Song L. H. Kim J. Sodek A. Koussevitzky S. (2009). Thiamin confers enhanced tolerance to oxidative stress in *Arabidopsis*. *Plant Physiol.* 151 421–432. 10.1104/pp.109.140046 19641031 PMC2735988

[B185] Ueland P. M. Mccann A. Midttun O. Ulvik A. (2017). Inflammation, vitamin B6 and related pathways. *Mol. Aspects Med.* 53 10–27. 10.1016/j.mam.2016.08.001 27593095

[B186] USDA (2016). *US Department of Agriculture, Agricultural Research Service, Nutrient Data Laboratory. USDA National Nutrient Database for Standard Reference, Release* 28 Beltsville, MD: US Department of Agriculture.

[B187] Van de Poel B. Van Der Straeten D. (2014). 1-aminocyclopropane-1-carboxylic acid (ACC) in plants: more than just the precursor of ethylene! *Front*. *Plant Sci.* 5:640. 10.3389/fpls.2014.00640 25426135 PMC4227472

[B188] Van Wilder V. De Brouwer V. Loizeau K. Gambonnet B. Albrieux C. Van Der Straeten D. (2009). C1 metabolism and chlorophyll synthesis: the Mg-protoporphyrin IX methyltransferase activity is dependent on the folate status. *New Phytol.* 182 137–145. 10.1111/j.1469-8137.2008.02707.x 19076298

[B189] Vanderschuren H. Boycheva S. Li K. T. Szydlowski N. Gruissem W. Fitzpatrick T. B. (2013). Strategies for vitamin B6 biofortification of plants: a dual role as a micronutrient and a stress protectant. *Front. Plant Sci.* 4:143. 10.3389/fpls.2013.00143 23734155 PMC3659326

[B190] Vanderstraeten L. Van Der Straeten D. (2017). Accumulation and transport of 1-aminocyclopropane-1-carboxylic acid (ACC) in plants: current status, considerations for future research and agronomic applications. *Front. Plant Sci.* 8:38. 10.3389/fpls.2017.00038 28174583 PMC5258695

[B191] Vandevijvere S. Amsalkhir S. Van Oyen H. Moreno-Reyes R. (2012). Determinants of folate status in pregnant women: results from a national cross-sectional survey in Belgium. *Eur. J. Clin. Nutr.* 66 1172–1177. 10.1038/ejcn.2012.111 22909577

[B192] Wachter A. Tunc-Ozdemir M. Grove B. C. Green P. J. Shintani D. K. Breaker R. R. (2007). Riboswitch control of gene expression in plants by splicing and alternative 3’ end processing of mRNAs. *Plant Cell* 19 3437–3450. 10.1105/tpc.107.053645 17993623 PMC2174889

[B193] Wagner S. Bernhardt A. Leuendorf J. E. Drewke C. Lytovchenko A. Mujahed N. (2006). Analysis of the *Arabidopsis rsr4-1/pdx1-3* mutant reveals the critical function of the PDX1 protein family in metabolism, development, and vitamin B6 biosynthesis. *Plant Cell* 18 1722–1735. 10.1105/tpc.105.036269 16766694 PMC1488916

[B194] Waller J. C. Alvarez S. Naponelli V. Lara-Nunez A. Blaby I. K. Da Silva V. (2010). A role for tetrahydrofolates in the metabolism of iron-sulfur clusters in all domains of life. *Proc. Natl. Acad. Sci. U.S.A.* 107 10412–10417. 10.1073/pnas.0911586107 20489182 PMC2890791

[B195] Wang H. C. De Steur H. Chen G. Zhang X. T. Pei L. J. Gellynck X. (2016). Effectiveness of folic acid fortified flour for prevention of neural tube defects in a high risk region. *Nutrients* 8:152. 10.3390/nu8030152 27005659 PMC4808880

[B196] Wang M. Goldman I. L. (1996). Phenotypic variation in free folic acid content among F-1 hybrids and open-pollinated cultivars of red beet. *J. Am. Soc. Hortic. Sci.* 121 1040–1042.

[B197] Watanabe S. Ohtani Y. Tatsukami Y. Aoki W. Amemiya T. Sukekiyo Y. (2017). Folate biofortification in hydroponically cultivated spinach by the addition of phenylalanine. *J. Agric. Food Chem.* 65 4605–4610. 10.1021/acs.jafc.7b01375 28548831

[B198] West K. P. Jr. Gernand A. Sommer A. (2007). “Vitamin A in nutritional anemia,” in *Nutritional Anemia* eds Kraemer K. Zimmerman M. B. (Basel: Sight and Life Press) 133–153.

[B199] Williams J. Mai C. T. Mulinare J. Isenburg J. Flood T. J. Ethen M. (2015). Updated estimates of neural tube defects prevented by mandatory folic acid fortification - United States, 1995-2011. *MMWR Morb. Mortal. Wkly. Rep.* 64 1–5. 25590678 PMC4584791

[B200] Win A. Z. (2016). Micronutrient deficiencies in early childhood can lower a country’s GDP: the Myanmar example. *Nutrition* 32 138–140. 10.1016/j.nut.2015.06.011 26421387

[B201] Yallew W. Bamlet W. R. Oberg A. L. Anderson K. E. Olson J. E. Sinha R. (2017). Association between alcohol consumption, folate intake, and risk of pancreatic cancer: a case-control study. *Nutrients* 9:E0448. 10.3390/nu9050448 28468303 PMC5452178

[B202] Yee W. S. Aziz S. D. A. Yusof Z. N. B. (2016). Osmotic stress upregulates the transcription of thiamine (vitamin B1) biosynthesis genes (*THIC* and *THI4*) in oil palm (*Elaeis guineensis*). *Afr. J. Biotechnol.* 15 1566–1574. 10.5897/AJB2016.15222

[B203] Youngblood M. E. Williamson R. Bell K. N. Johnson Q. Kancherla V. Oakley G. P. (2013). 2012 Update on global prevention of folic acid-preventable spina bifida and anencephaly. *Birth Defects Res. A Clin. Mol. Teratol.* 97 658–663. 10.1002/bdra.23166 24000219

[B204] Zaganjor I. Sekkarie A. Tsang B. L. Williams J. Razzaghi H. Mulinare J. (2015). Describing the global burden of neural tube defects: a systematic literature review. *Birth Defects Res. A Clin. Mol. Teratol.* 103 418–418. 10.1371/journal.pone.0151586 27064786 PMC4827875

[B205] Zeeman S. C. Thorneycroft D. Schupp N. Chapple A. Weck M. Dunstan H. (2004). Plastidial α-glucan phosphorylase is not required for starch degradation in *Arabidopsis* leaves but has a role in the tolerance of abiotic stress. *Plant Physiol.* 135 849–858. 10.1104/pp.103.032631 15173560 PMC514120

[B206] Zeng R. Xu C. H. Xu Y. N. Wang Y. L. Wang M. (2015). The effect of folate fortification on folic acid-based homocysteine-lowering intervention and stroke risk: a meta-analysis. *Public Health Nutr.* 18 1514–1521. 10.1017/S1368980014002134 25323814 PMC10271370

[B207] Zhao R. B. Gao F. Goldman I. D. (2001). Molecular cloning of human thiamin pyrophosphokinase. *Biochim. Biophys. Acta* 1517 320–322. 10.1016/S0167-4781(00)00264-5 11342117

[B208] Zhao Y. D. (2010). Auxin biosynthesis and its role in plant development. *Annu. Rev. Plant Biol.* 61 49–64. 10.1146/annurev-arplant-042809-112308 20192736 PMC3070418

[B209] Zhou H. R. Zhang F. F. Ma Z. Y. Huang H. W. Jiang L. Cai T. (2013). Folate polyglutamylation is involved in chromatin silencing by maintaining global DNA methylation and histone H3K9 dimethylation in *Arabidopsis*. *Plant Cell* 25 2545–2559. 10.1105/tpc.113.114678 23881414 PMC3753382

