# Supplementary material for: Toward Eradication of B-Vitamin Deficiencies: Considerations for Crop Biofortification
Source: Front Plant Sci. 2018 Apr 6;9:443. doi: 10.3389/fpls.2018.00443 (PMC5897740; doi:10.3389/fpls.2018.00443)
Supplement: TABLE S1 — An overview of the abbreviations. [file Table_1.docx]

| Full name | Abbreviation |
| --- | --- |
| AMINODEOXYCHORISMATE SYNTHASE | ADCS |
| 5-aminoimidazole ribonucleotide | AIR |
| Cauliflower mosaic virus | CaMV |
| Dihydrofolate | DHF |
| DIHYDROFOLATE REDUCTASE/THYMIDYLATE SYNTHASE | DHFR-TS |
| DIHYDROFOLATE SYNTHETASE | DHFS |
| FOLYLPOLYGLUTAMATE SYNTHETASE | FPGS |
| Glyceraldehyde 3-phosphate | G3P |
| Combined enhancement of GTPCHI and ADCS activty (metabolic engineering) | GA-strategy |
| Green fluorescent protein | GFP |
| GTP CYCLOHYDROLASE I | GTPCHI |
| Genome wide association study | GWAS |
| 4-methyl-5-β -hydroxyethylthiazole phosphate | HET-P |
| 6-hydroxymethyldihydropterin | HMDHP |
| 4-amino-2-methyl-5-hydroxymethylpyrimidine phosphate | HMP-P |
| HMDHP PYROPHOSPHOKINASE/DIHYDROPTEROATE SYNTHASE | HPPK/DHPS |
| Micronutrient malnutrition | MNM |
| METHYLENE-THF DEHYDROGENASE/METHENYL-THF CYCLOHYDROLASE | MTHFD1 |
| Nicotinamide adenine dinucleotide | NADH |
| Nicotinamide adenine dinucleotide phosphate | NADPH |
| Neural tube defect | NTD |
| α-KETO-GLUTARATE DEHYDROGENASE | OGDH |
| *para*-aminobenzoate | *p*-ABA |
| PYRUVATE DEHYDROGENASE | PDH |
| Pyridoxal phosphate synthase protein | PDX1 |
| B6 biosynthesis glutaminase | PDX2 |
| PMP/PNP OXIDASE | PDX3 |
| Pyridoxal | PL |
| Pyridoxal 5’-phosphate | PLP |
| pyridoxal reductase | PLR1 |
| Pyridoxamine | PM |
| Pyridoxine | PN |
| Quantitative trait locus | QTL |
| Ribose 5’-phosphate | R5P |
| Recommended daily allowance | RDA |
| Reactive oxygen species | ROS |
| REDUCED SUGAR RESPONSE | RSR4-1 |
| Salicylic acid | SA |
| S-adenosylmethionine | SAM |
| Systemic acquired resistance | SAR |
| Sustainable Development Goal | SDG |
| Sudden infant death syndrome | SIDS |
| SALT OVERLY SENSITIVE 4 | SOS4 |
| Tricarboxylic acid | TCA |
| HMP-P KINASE/TMP PYROPHOPHORYLASE | TH1 |
| TMP PHOSPHATASE/PALE GREEN1 | TH2 |
| Tetrahydrofolate | THF |
| HET-P SYNTHASE | THI1 |
| HMP-P SYNTHASE | THIC |
| TRANSKETOLASE | TK |
| Thiamin monophosphate | TMP |
| THIAMIN PYROPHOSPHOKINASE | TPK |
| Thiamin pyrophosphate | TPP |
| Wernicke’s encephalopathy | WE |
